# Generating a Generation of Proteasome Inhibitors: From Microbial Fermentation to Total Synthesis of Salinosporamide A (Marizomib) and Other Salinosporamides

**DOI:** 10.3390/md8040835

**Published:** 2010-03-25

**Authors:** Barbara C. Potts, Kin S. Lam

**Affiliations:** Nereus Pharmaceuticals, Inc., 10480 Wateridge Circle, San Diego, CA 92121, USA

**Keywords:** salinosporamide A, marizomib, NPI-0052, proteasome inhibitor, total synthesis, semi-synthesis, natural products chemistry, fermentation, mutasynthesis, precursor-directed biosynthesis, structure-activity relationships, analogs

## Abstract

The salinosporamides are potent proteasome inhibitors among which the parent marine-derived natural product salinosporamide A (marizomib; NPI-0052; **1**) is currently in clinical trials for the treatment of various cancers. Methods to generate this class of compounds include fermentation and natural products chemistry, precursor-directed biosynthesis, mutasynthesis, semi-synthesis, and total synthesis. The end products range from biochemical tools for probing mechanism of action to clinical trials materials; in turn, the considerable efforts to produce the target molecules have expanded the technologies used to generate them. Here, the full complement of methods is reviewed, reflecting remarkable contributions from scientists of various disciplines over a period of 7 years since the first publication of the structure of **1.**

## 1. Introduction

The ubiquitin and proteasome dependent proteolytic system (UPS) is the major pathway for regulated protein degradation in eukaryotic cells [1,2]. Central to the UPS is the 26S proteasome, a 2.5 MDa multi-catalytic enzyme complex that houses a 700 kDa proteolytic 20S core particle in which protein substrate hydrolysis is executed. Substrates for this non-lysosomal protein degradation pathway include misfolded and defective proteins, as well as others that are selectively polyubiquitin-tagged and targeted for degradation by the UPS [1–3]. Proteasome structure, function, and the impact of proteasome inhibitors as biochemical tools and therapeutic agents have been extensively reviewed [1–6]. In addition to providing a mechanism for cellular protein quality control, the UPS facilitates essential processes ranging from antigen processing to signal transduction, cell cycle control, cell differentiation and apoptosis [1–4]. These critical functions, together with the ubiquitious nature of the proteolytic 20S core particle, suggest a wealth of potential applications for proteasome inhibitors ranging from crop protection [7] and antiparasitics [8] to new therapies for inflammation [9] and autoimmune diseases [4], with demonstrated utility in the treatment of cancer [4,5,10–14].

**Figure 1:**
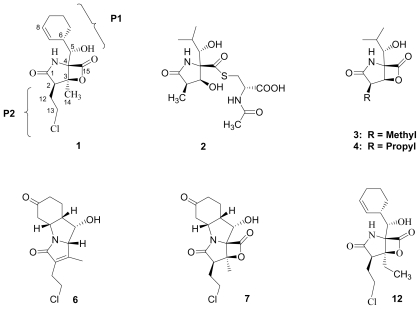
Structures of salinosporamide A (**1**), lactacystin (**2**), omuralide (**3**), PS-519 (**4**), salinosporamide C (**6**) and its hypothetical precursor (**7**), and salinosporamide I (**12**).

The proteasome's impact on diverse and essential cellular processes stems directly from its core function, *i.e.*, the proteolysis of a wide variety of target proteins. In turn, inhibiting proteasome activity has important downstream consequences that can be used to advantage in tumor cells, for example, the stabilization of proapoptotic proteins (e.g., p53, Bax, IκB) and the reduction of some antiapoptotic proteins (e.g., Bcl-2, NF- B), collectively inducing a proapoptotic state [4,5]. These and other findings provided strong rationale for targeting the proteasome for the treatment of cancer, an approach which received initial validation through Food and Drug Administration (FDA) approval of bortezomib [(*R*)-3-methyl-1-((*S*)-3-phenyl-2-(pyrazine-2-carboxamido)propanamido)butylboronic acid); PS-341; Velcade^®^] for the treatment of relapsed and relapsed/refractory multiple myeloma (MM) in 2003 [10,11]. Since that time, structurally unique proteasome inhibitors with the potential to treat patients that had failed or were not candidates for treatment with bortezomib have entered clinical trials [5]. One such agent is the marine-derived natural product salinosporamide A (marizomib; NPI-0052; **1**) (Figure 1) [15]. Accounts of its discovery and development have recently been reported [16,17] along with extensive preclinical indicators of strong clinical potential [5,12,13,16–22].

Identification and optimization of new inhibitors have benefited from knowledge of proteasome structure and biology; conversely, new proteasome inhibitors have contributed to the understanding of proteasome structure and function (for reviews, see [3,4,6]). The 26S proteasome comprises one or two 19S regulatory caps and a cylindrical 20S core particle housing three pairs of proteolytic subunits, β5, beta;2 and beta;1. These three subunit types have been ascribed chymotrypsin-like (CT-L), trypsin-like (T-L) and PGPH or caspase-like (C-L) activities based on their substrate preferences, and work in concert to degrade polyubiquitin-tagged proteins into small peptides. Substrate binding entails recognition of amino acid side chains (P1–Pn) by sequential binding pockets (S1–Sn) proximal to the enzyme active site, in analogy with other proteases. The S1 “specificity pocket” immediately adjacent to the active site largely confers the CT-L, T-L, and C-L sites with their preferential (albeit non-exclusive) binding to hydrophobic, positively-, and negatively-charged residues, respectively. Once bound, hydrolysis of the substrate peptide bond adjacent to S1 is catalyzed by the *N*-terminal threonine residue (Thr1), classifying the 20S proteasome among the *N*-terminal hydrolase family of enzymes. Thr1NH_2_ putatively acts as the general base, catalyzing Thr1OH^γ^ nucleophilic addition to the sessile substrate peptide bond to initiate bond cleavage. Based on this mechanism, it is perhaps not surprising that bortezomib and many other known proteasome inhibitors comprise peptides that are derivatized with reactive functional groups at the C-terminus, enabling formation of covalent adducts with Thr1 [3,4,6].

**Figure 2:**
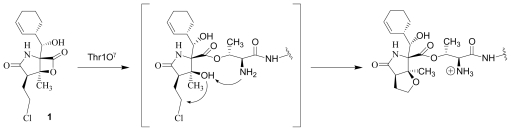
Mechanism of 20S proteasome inactivation by **1**. The β-lactone of the inhibitor acylates Thr1O^γ^, followed by displacement of chloride to form a 5-membered cyclic ether ring [35].

Despite the rational basis for peptidyl inhibitors, the structurally unique and terrestrially-derived microbial natural product lactacystin (**2**), comprising a γ-lactam substituted with a thioester and an isopropylcarbinol [23,24] (Figure 1), was found to specifically target the proteasome [25]. Lactacystin undergoes *in situ* transformation to the corresponding β-lactone known as “clastolactacystin-β-lactone” or “omuralide” (**3**), which represents the active species that acylates Thr1Oγ in the proteasome active site [25–28]. The evolution of **2** and **3** as biochemical tools that played pivotal roles in identifying the proteasome catalytic residues and enhancing general understanding of proteasome biology marked the birth of the β-lactone-γ-lactam family of proteasome inhibitors. Moreover, the structures of **2** and **3** offered attractive synthetic targets that inspired elegant and inventive strategies (for reviews, see [29–32]). Although **3** has not been developed as a therapeutic agent, its affinity and specificity for the proteasome demonstrated that peptidyl inhibitors can be challenged by densely functionalized lower molecular weight ligands of the β-lactone-γ-lactam family. In fact, the close structural analog PS-519 (**4**) (Figure 1) was evaluated in Phase I clinical trials based on preclinical data demonstrating neuroprotective efficacy in a preclinical model of cerebral eschaemia [33]. Then, in a timely 2003 publication, Fenical and coworkers reported that the marine actinomycete *Salinispora tropica* produced the potent and structurally novel proteasome inhibitor salinosporamide A (marizomib; NPI-0052; **1**; Figure (1) [15]. The fused bicyclic ring system of **1** revealed its structural relationship to **3** and suggested that the two molecules may share a common molecular target. This hypothesis was confirmed by assaying the two compounds for inhibition of purified 20S proteasome CT-L activity, and also established the enhanced potency of **1** (IC_50_ = 1.3 nM) versus **3** (IC_50_ = 49 nM) [15]. Moreover, **3** inhibited only CT-L activity while **1** inhibited all three proteolytic activities (CT-L, T-L, and C-L) [13,34]. *In vitro* cytotoxicity assays for **1** revealed IC_50_ values in the nM range against a panel of cancer cell lines [13,15,34], including MM, where proteasome inhibitors have shown clinical benefit [10,11]. Again, **1** (MM cell line RPMI 8226, IC_50_ = 8 nM) exhibited enhanced potency over **3** (RPMI 8226, IC_50_ = 3300 nM) [34]. The enhanced activity of **1** is rooted in its unique structure. While related to **3** by virtue of the shared β-lactone-γ-lactam core structure, **1** is distinguished by chloroethyl, methyl, and cyclohex-2-enylcarbinol substituents at the C-2, C-3 and C-4 positions, respectively, which give rise to specific and mechanistically important interactions within the proteasome active site that include recognition of the cyclohexenyl group by the S1 specificity pocket and acylation of the catalytic Thr1O^γ^ by the β-lactone followed by chloride displacement, rendering the ligand irreversibly bound (Figure 2) [35]. Recognizing the potential for the unique properties of **1** to translate into therapeutic benefit, the compound was licensed from the University of California, San Diego (UCSD) to Nereus Pharmaceuticals, San Diego, CA [16,17]. Intensive preclinical development included evaluation of marizomib in various solid tumor and hematological cancer models [12,13,16–22]. A human MM xenograft model in immunodeficient mice demonstrated efficacy after twice weekly IV (0.15 mg/kg) or oral (0.25 mg/kg) administration. Specifically, **1** inhibited MM tumor growth *in vivo* and prolonged survival, without the reoccurrence of tumor in 57% of mice. With respect to proteasome inhibition, treatment with **1** resulted in sustained inhibition of the CT-L, T-L and C-L activities in packed whole blood, a profile that was distinct from bortezomib. Moreover, **1** induced apoptosis in MM cells that were resistant to conventional and bortezomib therapies, without affecting normal lymphocyte viability, and did not affect the viability of MM patient-derived bone marrow stromal cells [13]. Interestingly, the two structurally distinct proteasome inhibitors, marizomib (**1**) and bortezomib, triggered differential apoptotic signaling pathways, suggesting a rationale for evaluating them in combination; indeed, combinations of low doses of the two agents triggered synergistic anti-MM activity [12,13,18]. These findings established the basis for a clinical development program, and an Investigational New Drug (IND) application was filed with the FDA in 2005 [16,17]. Strong preclinical indicators were also observed in leukemia cells [19–21], including synergistic cytotoxicity with histone deacetylase inhibitors (HDACi) [20,21], which provided rationale for ongoing clinical trials combining **1** with the HDACi, vorinostat [36]. In addition to promising results in hematological cancer models, oral administration of **1** improved tumoricidal response to multidrug treatment in a colon cancer xenograft model [22]. These and other studies suggested that **1** may be efficacious against hematological and solid tumors either as a single agent, and/or in combination with biologics, chemotherapeutics and targeted therapeutic agents [17]. At the time of writing, marizomib is being evaluated in several concurrent phase 1 clinical trials in patients with multiple myeloma, lymphomas, leukemias and solid tumors, including those that have failed bortezomib treatment, as well as patients with diagnoses where other proteasome inhibitors have not demonstrated efficacy [5,12,16,17,36–38].

The structure of **1**, together with its enhanced potency and therapeutic potential, sparked intense interest from the synthetic organic chemistry community (for an earlier review, see [32]). While strategies towards its *de novo* total synthesis reaped some benefits from the teachings of omuralide (vide supra), the more reactive functional groups of **1**, along with the additional stereocenter at C-6, added a new level of complexity that raised the bar of the synthetic challenge. This was answered with several enantioselective total synthetic routes [39–43] and complemented by a growing number of racemic strategies [44,45] and formal syntheses [46–50] (see Section 6. Total Synthesis of **1**). Nevertheless, *S. tropica* remains the most efficient producer of **1**. In a key demonstration of the industrial potential of marine microbiology, clinical supplies of **1** are being manufactured through a robust saline fermentation process [16,17] (see Section 2.2. Fermentation Optimization of **1** to Clinical Trials Materials). In parallel, *S. tropica* was further exploited in several important ways: (i) structurally related natural products were identified [51–53] (see Section 2.1 Natural Products of S. tropica); (ii) modified media and precursor-directed biosynthesis gave rise to new chemical entities, altered the ratios of secondary metabolites, and offered insights into the biosynthetic pathways of **1** and analogs [52,54–58] (see Section 2.3 Products of Precursor-Directed Biosynthesis); (iii) access to large quantities of **1** through fermentation enhanced its utility as a precursor for semi-synthesis [34,52,59–61] (see Section 4. Products of Semi-Synthesis); (iv) its genome was sequenced [62]; and (v) knockout mutants were generated, opening the door to bioengineered products [63–66] (see Section 2.4 Products of Mutasynthesis). Here, we capture the full complement of methods for generating a generation of proteasome inhibitors in the salinosporamide family that have been developed by microbiologists and organic chemists working collaboratively or independently. The collective body of work reflects enormous progress over a period of 7 years since the first publication of **1** by Fenical and coworkers [15].

**Figure 3:**
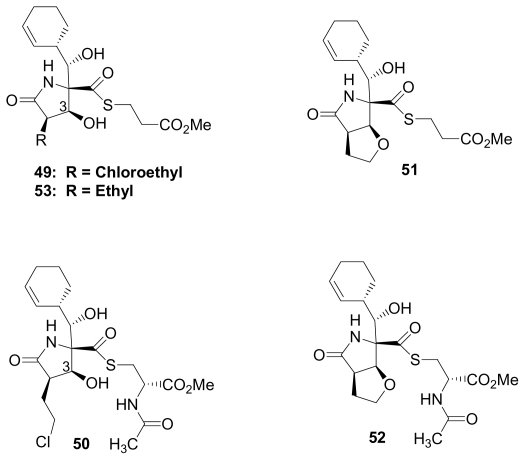
Thioester derivatives of **1** and **5**.

**Scheme 1:**
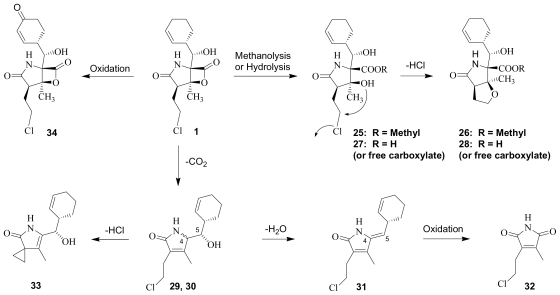
Degradation pathways for **1**: Hydrolysis, oxidation, and decarboxylation.

**Scheme 2:**
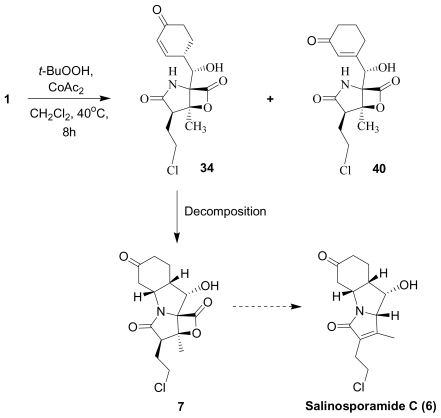
Oxidation of **1** gave a mixture of cyclohexenones **34** and **40**. Decomposition of **34** gave **7**, the hypothetical precursor to salinosporamide C (**6**).

**Scheme 3:**
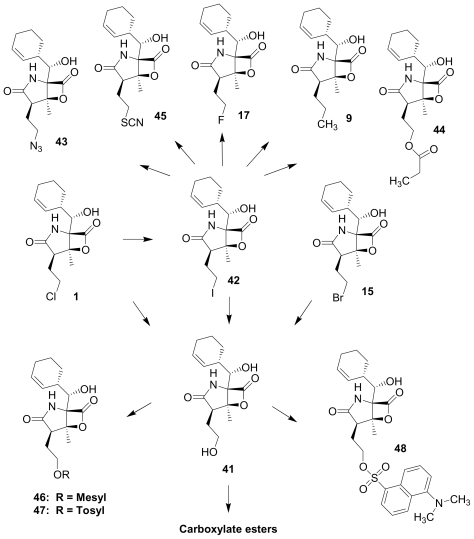
Overview of the semi-synthesis of P2 analogs.

**Scheme 4:**
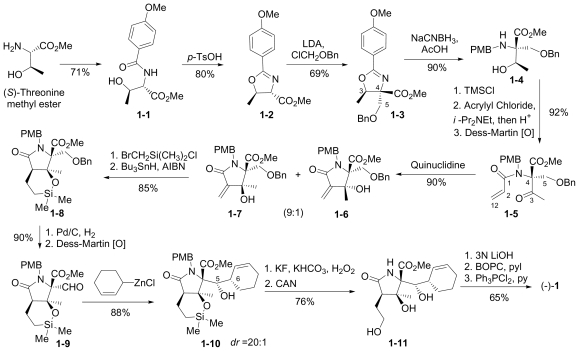
Corey and coworkers’ synthesis of (-)-**1** from L-threonine methyl ester [39].

**Scheme 5:**
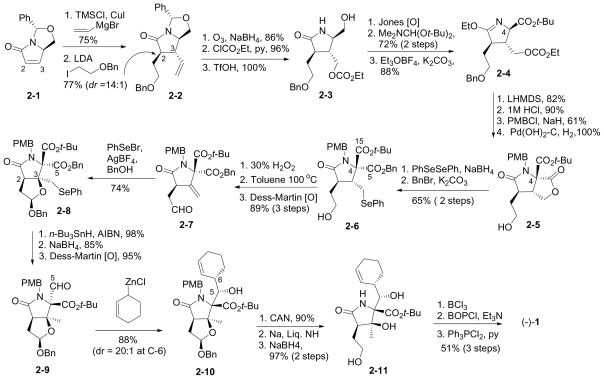
Danishefsky and Endo synthesis of (-)-**1** from a pyroglutamate derivative of L-glutamic acid [40].

**Scheme 6:**
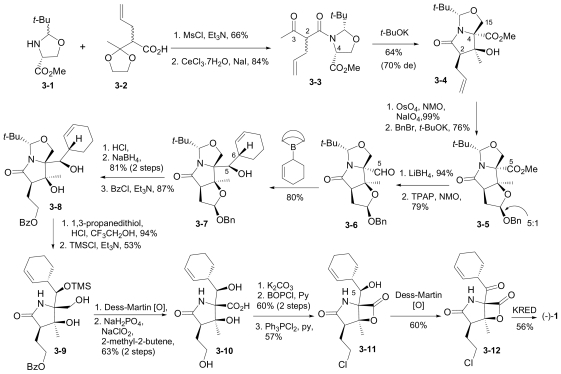
Nereus synthesis of (-)-**1** from D-serine [41].

**Scheme 7:**
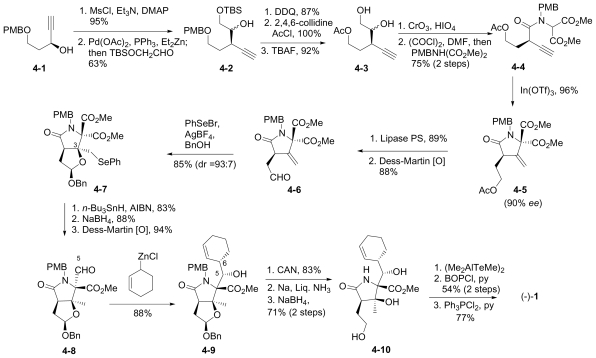
Hatakeyama and coworkers’ synthesis of (-)-**1** based on an indium-catalyzed Conia-ene reaction [42].

**Scheme 8:**
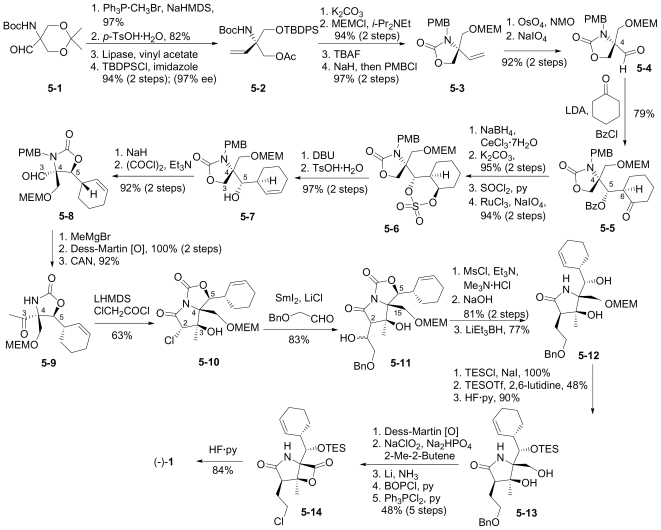
Omura and coworkers’ synthesis of (-)-**1** through novel cyclohexene construction [43].

**Scheme 9:**
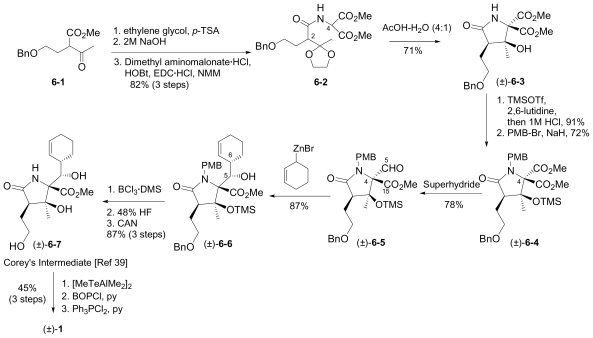
Pattenden and coworkers’ synthesis of (±)-**1** [44,104].

**Scheme 10:**
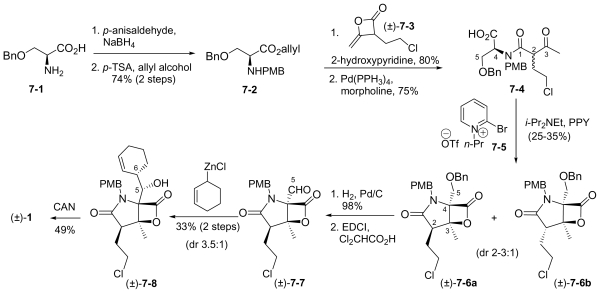
Romo and coworkers’ racemic synthesis of (±)-**1** [45].

**Scheme 11:**
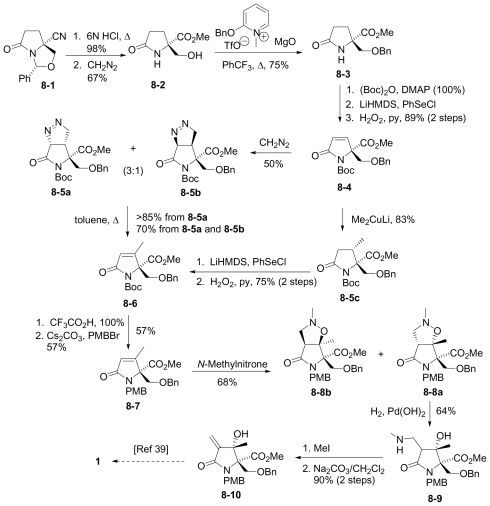
Langlois and coworkers’ stereoselective formal synthesis of **1**. Synthesis of **8-2** from **8-1** was performed according to [107]. **8-10** was synthesized from **8-2** as described in [47,106].

**Scheme 12:**
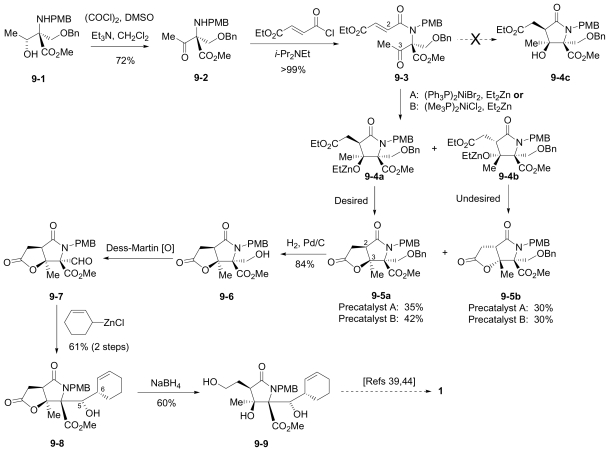
Lam and coworkers’ formal synthesis of **1** using a nickel-catalyzed reductive aldol cyclization-lactonization as a key step [47].

**Scheme 13:**
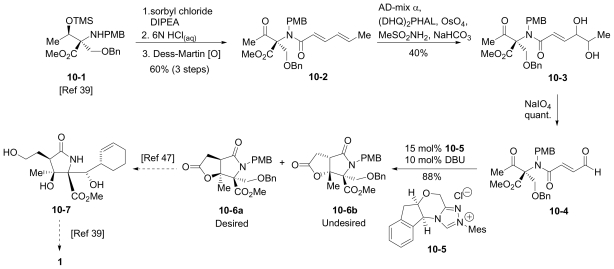
Bode and Struble formal synthesis of **1** *via* NHC-catalyzed intramolecular lactonization [48].

**Scheme 14:**
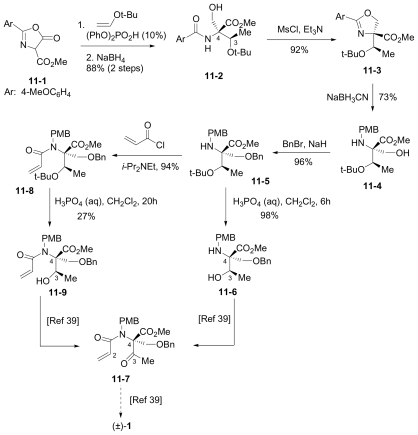
Tepe and Mosey formal synthesis of (±)-**1** [49].

**Scheme 15:**
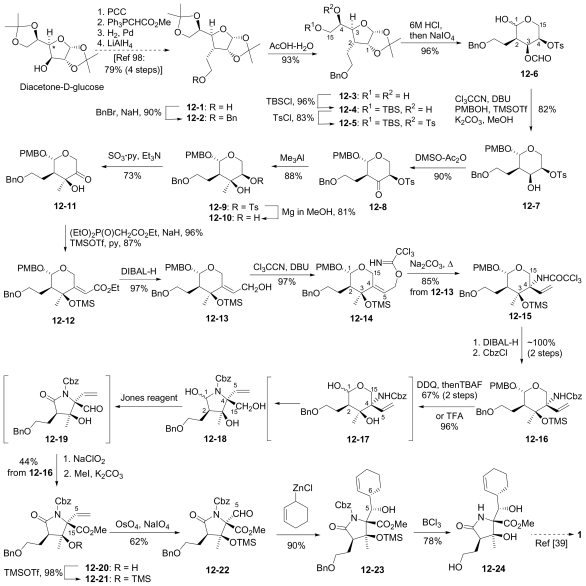
Chida and coworkers’ formal synthesis of (-)-**1** from D-glucose [50].

As a guide to the reader, we refer to Figure 1 and adopt the following nomenclature throughout this review. Based on crystallographic analysis of β-lactone-γ-lactam inhibitors in complex with the 20S proteasome [28,35,67] and the orientation of key substituents relative to those of peptidyl inhibitors, the C-4 substituent is referred to as the P1 residue, while the C-2 substituent is denoted P2 (despite its non-amino acid origins [68]). Other substituents and functional groups will be referred to by atom number according to Figure 1. All P1 and P2 analogs are captured in Tables 1 and 2, along with their published methods of production and their IC_50_ values for inhibition of purified 20S proteasome CT-L activity. Compounds that fall outside of these structural boundaries are captured in Figures 1 and 3 and Schemes 1–3. Finally, the synthetic routes for the total and formal synthesis of **1** are presented in Schemes 4–15. While the main focus of this review article is on methods of production, structure-activity relationship trends are briefly presented (see Section 5. Structure-Activity Relationships).

## 2. Natural and Unnatural Products of *S. tropica*

In this section, we focus on salinosporamides generated from *S. tropica*, including those isolated from wild type and genetically modified strains.

### 2.1. Natural Products of *S. tropica*

The genus *Salinispora* represents a group of taxonomically diverse actinomycetes that is widely distributed in ocean sediments [69,70]. The discovery of this marine taxon was part of a larger effort by Fenical and coworkers to explore the ocean as a source of new marine microbes that produce novel chemical entities with therapeutic potential. Strains representing three *Salinispora* species (*tropica*, *arenicola*, and *pacifica*) were isolated from samples collected in tropical and subtropical regions, and fermentation extracts produced from these strains gave rise to a high hit rate in anticancer and antibiotic screens. A detailed investigation of *S. tropica* ensued, which led to the discovery of **1** [15,16].

*S. tropica* was first isolated from a heat-treated marine sediment sample collected in the Bahamas. The potent biological activity of crude extracts obtained from shake-flask culture and solid phase extraction led to the bioassay guided fractionation and isolation of the major secondary metabolite salinosporamide A (**1**) by Feling *et al.* [15]. Structure elucidation revealed its dense functionality (Figure 1), including the fused bicyclic β-lactone-γ-lactam core reminiscent of omuralide (**3**) and 5 contiguous stereocenters (2*R*,3*S*,4*R*,5*S*,6*S*) that were unequivocally established by X-ray crystallography. Publication of initial findings on the source organism, structure and proteasome inhibitory profile of **1** in 2003 [15], when the proteasome was receiving considerable attention through positive clinical trials results with bortezomib [10,11], triggered a rigorous preclinical evaluation of **1** that formed the basis for ongoing clinical trials (vide supra).

Encouraged by the phylogenetic novelty of *Salinispora* and the exciting new chemistry exemplified by **1**, research on *S. tropica* continued at UCSD. A thorough evaluation of crude extracts resulted in the identification of deschloro analog salinosporamide B (**5**) (Table 1) [51]. Although less potent than **1** in terms of proteasome inhibition and cytotoxicity, **5** provided important mechanistic and biosynthetic insights: biochemical and structural biology studies of **5** in direct comparison with **1** highlighted the importance of the chlorine leaving group of the parent natural product for inducing irreversible binding to the proteasome [35,61], while precursor-directed biosynthesis demonstrated distinct origins for the C1/C2/C12/C13 carbons of **1** versus **5** [55]. Salinosporamide C (**6**), a tricyclic cyclohexanone derivative of **1**, was also isolated from the fermentation broth; while considered a natural product, rearrangement pathways from **1** involving the proposed β-lactone precursor **7** were envisioned (Figure 1) [51]. Moreover, **7** was subsequently isolated as a byproduct of chemical transformations of **1** under oxidative conditions (Macherla, Manam and Potts, unpublished observation; see Section 4. Products of Semi-synthesis). In addition to salinosporamides A–C, *S. tropica* crude extracts contained several products of β-lactone ring hydrolysis or decarboxylation. The ability to generate these compounds from **1** under conditions similar to those used during fermentation and extraction led to their assignment as degradants as opposed to natural products [51]. Nevertheless, these findings offered important insights into the reactivity of **1** (for structures and discussion, see Section 3. Products of Chemical Degradation).

The advancement of **1** into preclinical development at Nereus demanded a constant supply of pure compound, which further necessitated fermentation scale-up and process development (see Section 2.2. Fermentation Optimization of **1** to Clinical Trials Materials). Purification of **1** from larger scale crude extracts (8 g derived from 40 L of fermentation broth) facilitated the isolation and structural characterization of several less abundant congeners [52]. Most of these natural products represented modifications to P2 (Table 1), including salinosporamide D (**8**; P2 = methyl), the previously described salinosporamide B (**5**; P2 = ethyl), and salinosporamide E (**9**; P2 = propyl), the latter of which had first been identified by semi-synthesis [34]. Stereoisomers representing epimers at the C-2 position were also identified, including salinosporamides F (**10**) and G (**11**), the C-2 epimers of **1** and **8**, respectively [52]. Sampling and HPLC analysis of fermentation culture extracts over time indicated that the ratio of **1** to **10** was fairly constant throughout the fermentation cycle, suggesting that C-2 is not post-biosynthetically racemized. The corresponding diastereomer of salinosporamide B was not detected in the large scale crude extract but was identified in extracts obtained from modified fermentation conditions using NaBr-based media (see Section 2.3. Products of Precursor-Directed Biosynthesis). In addition to these P2 congeners, the large scale crude extract contained salinosporamide I (**12**), in which the methyl group at the C-3 ring junction is replaced with an ethyl group (Figure 1), and P1 analog salinosporamide J (**13**) (Table 2), reflecting dehydroxylation at C-5 [52].

Up to 2007, only natural products bearing a cyclohexenyl substituent at C-5 had been identified; specifically, congeners with an omuralide-like isopropyl group had not been reported. Nevertheless, the structural similarity between **1** and omuralide (**3**) inspired the total synthesis of ‘antiprotealide’ (**14**), a molecular hybrid in which the cyclohexenyl substituent of **1** is replaced with an isopropyl group, as contributed by Corey and coworkers in 2005 (Table 2) [71,72]. Then, in 2008, **14** was reported as product of bioengineering of *S. tropica* [64]. Meanwhile, **1** had advanced from preclinical to clinical development, and large scale production of clinical trials materials was undertaken at up to 1000 L scale (vide infra). Purification of **1** from 72 g of crude extract obtained from a 350 L fermentation broth generated side fractions that were enriched in salinosporamide B (**5**) and a new congener, which spectroscopic analysis revealed to be identical to antiprotealide [53]. While access to large scale fermentation extracts facilitated in its identification, analysis of crude extracts from shake flask cultures of three wild type *S. tropica* strains confirmed the production of **14** in quite reasonable titers of ~1 to 3 mg/L. These findings firmly established antiprotealide as a natural product of *S. tropica* [53]. Antiprotealide represents one of a limited number of cases in which a natural product was identified subsequent to its synthesis. Notably, the synthesis of antiviral agent 9-(β-D-arabinofuranosyl)adenine (Ara-A) [73] preceded the production of the same compound by fermentation of *Streptomyces antibioticus* [74].

The demonstration that wild type *S. tropica* strains produce antiprotealide, together with its close structural relationship to omuralide, begged the question of whether lactacystin-like analogs might also be natural products of *S. tropica*. Despite our thorough examination of *S. tropica* extracts, thioester analogs of the salinosporamides were not identified, nor have they been reported by other laboratories. In contrast, omuralide (**3**) is found in nature as its thioester precursor lactacystin (**2**) [23,24], while the structurally related cinnabaramides (P1 = cyclohexenylcarbinol; P2 = substituted or unsubstituted *n*-hexyl) are also found as either β-lactones or thioesters [7,75]. However, these terrestrially-derived natural products do not bear halogen leaving groups at the P2 position. Semi-synthetic analogs of the marine-derived natural product **1** confirmed that the thioester form is prone to premature triggering of chloride displacement, rending the molecule significantly less active and offering no apparent advantage to the producing organism [52] (see Section 4. Products of Semi-Synthesis).

### 2.2. Fermentation Optimization of **1** to Clinical Trials Materials

In order to execute a successful preclinical development program, a reliable source of high purity drug substance (*i.e.*, “active pharmaceutical ingredient” (API)) is required. The original fermentation conditions and the production strain (*S. tropica* CNB476) transferred from Fenical’s research group at Scripps Institution of Oceanography, UCSD, afforded the production of a few mg per liter of **1** in shake flask cultures. The original seed and production media contained numerous animal-derived media components and natural seawater that cannot be used to manufacture the API under current Good Manufacturing Practice (cGMP). Extensive fermentation development to replace the non-compliant media components and improve production was carried out at Nereus Pharmaceuticals. We successfully replaced seawater with a commercially available synthetic sea salt, Instant Ocean, for the production of **1**. We also replaced all animal-derived nutrients with plant-derived nutrients to meet the FDA requirement. The yield improvement processes are summarized in Table 3 and discussed below.

It has been well documented that the addition of resins to the fermentations of reactive and/or highly potent secondary metabolites leads to increases in production of these metabolites [76–79]. The key to the initial success of yield improvement of **1** was the addition of solid resins to the production culture (Table 3; step 1). The inherent instability of the β-lactone ring of **1** in aqueous solution [80], such as in the submerged saline fermentation, was overcome by addition of solid resin to the fermentation in order to bind and capture **1**. The addition of resin to the production culture led to an 18-fold increase in yield in a preliminary study (Table 3). Further investigation of the resin stabilization effect on **1** using production strain NPS21184 (see below) established the conditions for the large-scale resin addition process [81].

Wild type strain often contains a heterogeneous population of cells that have different productivity. A simple experiment involves spreading the wild type strain on agar plates to obtain single colonies, comparing the productivity of the single colonies, and selecting the colony with the best productivity and/or characteristics for further studies. The second key yield improvement for **1** was the isolation of *S. tropica* strain NPS21184, a single colony isolate directly derived from strain CNB476 without mutation and genetic manipulation (Table 3; step 4). Besides supporting higher production of **1**, strain NPS21184 produces three-fold less of deschloro analog **5** than the parent strain CNB476. This is beneficial given that this interfering analog must be removed during API purification.

When developing an industrial fermentation process, designing the fermentation medium is of critical importance. The fermentation medium affects the product yield and volumetric productivity, and also needs to comply with cGMP guidelines set by FDA. Media formulation studies (Table 3; steps 3 and 5) were successfully carried out to replace natural seawater and animal-derived media components with media components that are acceptable for cGMP manufacturing. Furthermore, additional yield improvement was achieved *via* media formulation studies. A greater than 100-fold increase in the production of **1** in shake flask culture was obtained after the above yield improvement processes with a production titer of 450 mg/L.

The production of **1** by marine actinomycete strain NPS21184 was carried out *via* a saline fermentation process. Saline fermentation poses a major challenge in scale-up since published literature suggested that the 316-type stainless steel fermentors commonly found in manufacturing facilities are not resistant to the corrosive effect of the saline media [82]. Using a 316 stainless steel B. Braun Biostat-C fermentor (42 L total volume), we developed a process to overcome the corrosive effect of saline fermentation media based on this stainless steel fermentor. The foaming, aeration and agitation issues associated with the scale-up production of **1** in fermentors were also addressed using the B. Braun Biostat-C fermentor.

We successfully transferred the yield improvement conditions developed in shake flasks to a laboratory fermentor as shown in Table 3. A titer of 360 mg/L for **1** was achieved in the 42 L laboratory fermentor, which is lower than the maximum titer of 450 mg/L detected in shake flask. The discrepancy in titers is due to the foaming problem that occurred in the fermentor (but not in shake flask) when rich media containing high concentrations of starch and soy type products were used.

Marizomib (**1**) API is currently manufactured under cGMP through a robust saline fermentation process by *S. tropica* strain NPS21184 at two different contract manufacturing organizations. The final fermentation process development effort standardized parameters such as temperature exposure, operating parameters, cleaning and passivation to overcome the corrosive effect of saline fermentation, and was performed in 500–1500 L industrial stainless steel fermentors. This, together with careful design of the timing and method for introducing the resin to the production fermentor, resulted in production titers of 250–300 mg/L in 500–1500 L industrial fermentors. During the peak production cycle, the resin-bound drug is collected, filtered, extracted with ethyl acetate and concentrated for downstream processing (DSP) in an environment with appropriate containment for a high biological potency substance. To maintain optimal stability of **1**, all DSP steps are executed in non-aqueous solvent systems. The crude extracts from the resin undergo purification involving a highly effective silica gel flash chromatography step, which removes all unrelated substances as well as most congeners of **1**, such as the deschloro analog **5**. In fact, the purity of **1** increases from ~55% to ~95% (UV area by HPLC) after this single flash chromatography step. The resulting highly purified API obtained after flash chromatography may contain up to ~3% of diastereomeric impurity **10**. Using an evaporative crystallization process that exploits subtle solubility differences between **1** and **10**, this impurity is reduced to <1%, and **1** is isolated as a white crystallize solid. The final pharmaceutical grade cGMP marizomib API is obtained in >98% purity with overall ~50% recovery from the crude extract. Based on the potency of **1**, the production titer at fermentor scale and the recovery yield are adequate for both clinical development and commercial production. To the best of our knowledge, this represents the first manufacture of clinical trial materials by saline fermentation.

### 2.3. Products of Precursor-Directed Biosynthesis

Precursor-directed biosynthesis is the addition to the fermentation medium of an analog of a part of the secondary metabolite which the organism is then capable of incorporating into its enzymatic process to yield a modified metabolite. The production of new secondary metabolites using directed biosynthesis is an attractive, efficient and simple-to-use method that has wide application in the field of industrial secondary metabolite production [83–87]. We have successfully employed this technique in increasing the production of minor salinosporamides and generating novel salinosporamides in *S. tropica* fermentations (Table 4).

One of our key successes in applying this technique to generate novel salinosporamides was in developing the proper media to support the production of the novel analogs. In the NaCl-based medium, **1** is the major product of the fermentation with a titer of 277 mg/L (Table 4, condition 1). Several minor P2 analogs, such as **8** (P2 = methyl; 0.15 mg/L), **5** (P2 = ethyl; 4.4 mg/L) and **9** (P2 = propyl; 0.11 mg/L), are coproduced in the *S. tropica* NPS21184 fermentation (Table 4, condition 1). Replacing the NaCl-based medium with a NaBr-based medium produced a novel brominated analog (**15**) as the second major salinosporamide (19 mg/L) in the fermentation (Table 4, condition 2). The major salinosporamide produced was deschloro analog **5** (80 mg/L), while **1** was only a minor component (1.2 mg/L) in the fermentation (Table 4, condition 2) [54]. The increased production of **5** was accompanied by the presence of its C-2 epimer **16** [52].

We developed a Na_2_SO_4_-based medium with no discrete chloride ion added to this medium to suppress the production of **1** (Table 4, condition 3). The production of **1** was significantly reduced to 53 mg/L while the productions of **5**, **8** and **9** were increased by 64 to 127% (Table 4, condition 3). By feeding 1.5% NaBr to this Na_2_SO_4_-based medium, the production of bromosalinosporamide (**15**) was significantly enhanced and was the major salinosporamide produced in the fermentation (73.3 mg/L) (Table 4, condition 4). The production of **1** was further reduced to 18.7 mg/L in the NaBr-fed medium (Table 4, condition 4). The result from this feeding study confirmed that bromide ion enhanced the production of **5** by 3-fold with a production titer of 22.3 mg/L (Table 4, condition 4; Zhu and Lam, unpublished observations).

Incorporation of fluorine could not be achieved *via* the approach used to generate bromosalinosporamide, as NaF (1–2%) inhibits the growth of the organism [54]. Moreover, fluoride is not a substrate for the chlorinase enzyme that catalyzes the synthesis of the 5′-chloro-5′-deoxyadenosine (5′-ClDA) precursor to **1** [68]. Replacing the *salL* chlorinase gene by the *Streptomyces cattleya FlA* fluorinase gene in *S. tropica*, Eustáquio *et al.* demonstrated that the resulting *S. tropica salL*^–^ *FlA*^+^ mutant strain can accept fluoride as a substrate for the production of fluorosalinosporamide (**17**) at a concentration of 4 mg/L [66]. The semi-synthesis of **17** was reported by Manam *et al.* in 2008, but the yield was low [61] (see Section 4. Products of Semi-Synthesis). The production of **17** by mutasynthesis (feeding 5′-fluoro-5′-deoxyadenosine (5′-FDA) to a *salL*^–^ knockout mutant of *S. tropica;* see Section 2.4. Products of Mutasynthesis) was reported by Eustáquio and Moore [63], however, both the volumetric productivity (1.5 mg/L) and conversion yield (5%) were low. Another application of the Na_2_SO_4_-based medium is the production of **17** by feeding 0.025% 5′-FDA to the medium. We obtained a volumetric productivity of **17** at 55.8 mg/L with a conversion yield of 22% by precursor-directed biosynthesis in the Na_2_SO_4_-based medium (Table 4, condition 5) (Lam, Tsueng, Potts and Macherla, unpublished observation), significantly superior to the mutasynthesis method. Furthermore, **17** is a minor salinosporamide in the fermentation produced by the mutasynthesis approach. In contrast, **17** is the major salinosporamide in the fermentation of the Na_2_SO_4_-based medium produced by the precursor-directed biosynthesis approach.

Antiprotealide (**14**) is a molecular hybrid comprising the core structure of **1** with the omuralide (**3**)-derived isopropyl group in place of the cyclohexene ring. **14** was first characterized by Corey and coworkers as a synthetic analog [71,72] and then as an unnatural salinosporamide produced by a genetically engineered strain of *S. tropica* [64]. While McGlinchey *et al.* reported that the parent type strain *S. tropica* CNB440 did not produce **14**, ~0.5 mg/L was detected in the *S. tropica salX*^–^ mutant in which the pathway for the biosynthesis of the cyclohexenyl moiety (L-3-cyclohex-2′-enylalanine) of **1** had been inactivated. Feeding 0.38 mM L-leucine to the *S. tropica salX*^–^ fermentation increased the production of **14** by 2-fold to ~1 mg/L and established that L-leucine is the biosynthetic precursor of **14** [64] (see *2.4. Products of Mutasynthesis*). We observed the production of **14** in three wild type strains of *S. tropica*, including the type strain CNB440 with a production titer of 1.1 mg/L, thereby establishing for the first time that antiprotealide (**14**) is indeed a natural product [53] (see *2.1. Natural Products of S. tropica*). The best production of **14** was observed in *S. tropica* NPS21184, a single colony isolate derived directly from wild type strain CNB476 without any mutation or genetic modification, with the titer of 3.0 mg/L (Table 4, condition 1). We later demonstrated that feeding 1% L-leucine to the *S. tropica* NPS21184 fermentation increased the production of **14** by 3.7-fold to 11 mg/L (Table 4, condition 11; Tsueng and Lam, unpublished observation).

We also examined the effect of feeding 1% L-isoleucine to the *S. tropica* NPS21184 fermentation, which increased the production of **8** from 0.15 mg/L (Table 4, condition 1) to 4.63 mg/L while completely inhibiting the production of antiprotealide (Table 4, condition 12). The 31-fold increase in production of **8** in the L-isoleucine-fed culture might be due to the fact that L-isoleucine is the precursor of propionate [88], which in turn, is the precursor of the contiguous three-carbon unit C-1/C-2/C-12 of **8**. The above postulation was confirmed by feeding 1% propionate to the *S. tropica* NPS21184 fermentation, which led to a similar production of **8** as in the L-isoleucine-fed culture (Table 4, condition 7). We further demonstrated that [^13^C] propionate was incorporated into **8** (Tsueng, McArthur, Potts and Lam, unpublished observations).

Feeding 1% L-valine to the *S. tropica* NPS21184 fermentation increased the production of **5** from 4.4 mg/L (Table 4, condition 1) to 16.7 mg/L while the production of antiprotealide was completely inhibited (Table 4, condition 10). The 3.8-fold increase in production of **5** in the L-valine-fed culture might be due to the fact that L-valine is the precursor of butyrate [88], which in turn, is the precursor of the contiguous four-carbon unit C-1/C-2/C-12/C-13 of **5** [55]. The above postulation was confirmed by feeding 1% butyrate to the *S. tropica* NPS21184 fermentation, which led to a similar 4.3-fold increase and production of **5** at 19 mg/L as in the L-valine-fed culture (Table 4, condition 8). The production of **5** in the control culture and the butyrate-fed culture were significantly less than reported in our previous publication [55] because the NaCl-based medium used in this study contains cobalt chloride. We have demonstrated that cobalt and vitamin B_12_ inhibit the production of **5** [58]. Even though the absolute amounts of production of **5** in these two studies are different, the effect of butyrate in increasing the production of **5** is the same (4.3-fold versus 4.2-fold). In the earlier study, we also confirmed the incorporation of [U-^13^C_4_]butyrate into **5**; feeding sodium [U-^13^C_4_]butyrate to *S. tropica* cultures enhanced the production of **5** by over 300% while inhibiting production of **1** by over 25%. NMR analysis confirmed the incorporation of butyrate as a contiguous 4-carbon unit (C-1/C-2/C-12/C-13) into **5** but not **1**, providing the first direct evidence that the biosynthesis of **5** is distinct from **1** and that **5** is not a precursor of **1** [55]. The precursor for the chloroethyl group of **1** was subsequently identified as 5′-ClDA [68].

The production of **9** by *S. tropica* NPS21184 is extremely low at 0.11 mg/L, compared to the production of **1** at 277 mg/L in shake flask culture (Table 4, condition 1). Feeding 1% valerate to the *S. tropica* NPS21184 fermentation in the NaCl-based medium led to a 1,100-fold increase in production of **9** to 121 mg/L and concomitant decrease in the production of **1** by 53% to 131 mg/L (Table 4, condition 9). We demonstrated the incorporation of valerate labeled with deuterium into **9** at the contiguous five-carbon unit C-1/C-2/C-12/C-13/C-16 and thereby established that valerate is the precursor of **9**. Even though the production of **9** was increased by 1,100-fold and was similar to the production of **1** in the valerate-fed culture grown in NaCl-based medium, the isolation of **9** was a major challenge due to the close chromatographic elution profile of **9** and **1**. We overcame this purification challenge by feeding 1% valerate to the *S. tropica* NPS21184 fermentation in the Na_2_SO_4_-based medium. While there was only a 20% increase in the production of **9** in the Na_2_SO_4_-based medium, the production of **1** decreased to 45 mg/L due to the reduction of chloride ion in the Na_2_SO_4_-based medium. With a significant increase in the ratio of **9** to **1**, the purification of **9** from **1** can now be achieved (Tsueng, McArthur, Potts and Lam, unpublished observations).

The above account demonstrates that precursor-directed biosynthesis together with the use of proper media represents a powerful technique for increasing the production of minor salinosporamides and generating novel salinosporamides.

### 2.4. Products of Mutasynthesis

Mutasynthesis, or mutational biosynthesis, is a term originally defined by Nagaoka and Demain [89] and by Rinehart and Stroshane [90] for the concept that an exogenous moiety is needed for the synthesis of a secondary metabolite by a mutant of the producing organism. The mutant which requires this special nutrient to produce a product peculiar to that organism has been termed an “idiotroph” [89]. Application of mutasynthesis in generating novel analogs of different classes of medically important secondary metabolites has been well documented [91–95].

Bioinformatic analysis of the salinosporamide biosynthetic gene cluster (*sal*) from the genome sequence of *S. tropica* CNB440 [62] revealed a subset of genes that were subsequently exploited for the bioengineering of new analogs by Moore and coworkers. To eliminate production of the nonproteinogenic amino acid L-3-cyclohexen-2′-enylalanine precursor to the P1 substituent of **1**, the prephenate dehydratase homologue gene *salX* was targeted for genetic disruption *via* PCR-based mutagenesis. Fermentation of the *S. tropica salX*^–^ disruption mutant, complemented by feeding select substrate amino acid precursors (proteinogenic, nonproteinogenic, and synthetic), successfully generated several target P1 analogs, including alicyclics **18**–**21** bearing cyclohexyl, cyclopentenyl, cyclopentyl, and cyclobutyl groups, respectively; branched aliphatic antiprotealide (**14**); straight chain aliphatics **22** and **23**; and phenyl analog **24** [64,65]. Using a similar approach, Eustáquito and Moore targeted SalL [63]. SalL chlorinates *S*-adenosyl-L-methionine to produce 5′-ClDA, the C1/C2/C12/C13-Cl precursor of **1**, and does not accept fluorine as a substrate [68]. Thus, fluorosalinosporamide (**17**) was generated by feeding 5′-FDA to a *salL*^–^ knockout mutant of *S. tropica* that had lost the capacity to produce **1** [63]. Recently, Eustáquio *et al.* demonstrated that replacing the *salL* chlorinase gene in *S. tropica* with a *Streptomyces cattleya FlA* fluorinase gene resulted in an *S. tropica salL*^–^ *FlA*^+^ mutant strain that can accept fluoride as a substrate for the production of **17** at a concentration of 4 mg/L [66]. Fluorosalinosporamide has also been generated in low yield semi-synthetically [61] (vide infra), but now most successfully by directly feeding 5′-FDA to the wild-type *S. tropica* strain in a Na_2_SO_4_-based medium, as reported herein (see *2.3. Products of Precursor-Directed Biosynthesis*).

## 3. Products of Chemical Degradation

While highlighting chemical degradation in an account devoted to methods of preparing intact target molecules may seem unusual, knowledge of the mechanisms by which **1** is degraded led to the incorporation of appropriate precautionary measures and processes to circumvent or attenuate degradation during API manufacturing (vide supra), formulation development, and processing of blood samples for pharmacokinetic analysis. Moreover, the products of β-lactone ring opening effectively anticipated the chemical mechanism of inhibition of the 20S proteasome by **1** [34,35].

Chemical degradants of **1** are largely formed *via* β-lactone ring hydrolysis or decarboxylation, with oxidation of the cyclohexene ring occurring as a minor pathway (Scheme 1). Methanolysis of the β-lactone to the corresponding methyl ester **25** was noted in the original account of the discovery of **1** [15] and formally characterized by Williams *et al.* [51]. Unveiling of the C-3 tertiary alcohol upon cleavage of the β-lactone ring was followed by intramolecular nucleophilic displacement of chloride to give **26**. We subsequently reported the analogous carboxylic acids NPI-2054 (**27**) and NPI-2055 (**28**) as products of aqueous hydrolysis, which was highly accelerated in base but occurred very slowly in acid. These structures led us to propose that chloride elimination may occur subsequent to Thr1O^γ^ acylation at the proteasome active site (Figure 2) [34], which was later confirmed by crystallography of **1** in complex with the yeast 20S proteasome [35]. Detailed kinetic studies by Denora *et al.* [80] demonstrated that -lactone ring hydrolysis occurs *via* standard ester hydrolysis (as opposed to a carbonium ion mechanism) and is moderately buffer-catalyzed, pH-independent in the range of 1–5, and base-dependent above pH 6.5. A kinetic deuterium isotope effect showed that the rate-determining step involves only a single proton transfer, suggesting that the neighboring C-5OH (as opposed to a second water molecule) facilitates attack of water at the β-lactone ring. The subsequent nucleophilic displacement of chloride is also moderately buffer catalyzed. The data suggested that **27** exists in the carboxylate form above pH 4; at lower pH values, the (protonated) carboxylic acid is expected to inhibit further degradation: the rate of conversion from **27** to **28** was slowest in the pH range 1–3; a plateau or pH-independent region was observed at pH 4.5–6.5; and at pH > 7, the degradation rate increased with increasing pH [80]. While reactions in aqueous buffer cannot directly model the drug-enzyme complex, these findings are consistent with base (Thr1NH_2_) catalyzed nucleophilic displacement of the halide in the proteasome active site [35,67]. In fact, Thr1N is sufficiently basic to catalyze the unusual reaction of fluoride displacement from an sp3-carbon in the case of fluorosalinosporamide (**17**), which recently led us to propose a proteasome Thr1NH_3+_ pKa > 10 [67].

The second dominant mechanistic pathway for the degradation of **1** involves decarboxylation (Scheme 1). Three products of this pathway (**29**, **30** and **31**) were isolated from *S. tropica* crude extracts; direct conversion of **1** to these same products under pH conditions identical to those used during fermentation allowed them to be assigned as degradants as opposed to natural products [51]. It was proposed that the two diastereomers are generated with retention of configuration at C-5 (**29** and **30**) followed by dehydration to give **31**. During the course of our semi-synthetic studies, we frequently observed the formation of these same products at elevated pH, particularly in the presence of tertiary amines. The diasteromeric pair **29** and **30** could be purposefully generated in the presence of triethylamine in dichloromethane at 40 °C; concentration at elevated temperatures gave **31** as a byproduct. Low levels of these same degradants were also detected during cGMP stability studies of the parenteral Phase 1 cosolvent formulation of **1** [0.24 mg/mL in 98% propylene glycol, 2% ethanol], particularly under accelerated (elevated temperature) conditions (Manam, Macherla and Potts, unpublished observations). Overall, we observed that the C-4 diasteromers **29** and **30** formed first, followed by **31**, supporting the earlier suggestion that C-4/C-5 dehydration occurs as the final step in the degradation pathway. Degradant **32** was also detected during cosolvent formulation stability testing, and presumably results from further oxidative cleavage of the C-4/C-5 double bond of **31**. The relatively high UV extinction coefficient for these conjugated compounds overestimates their presence in samples when not corrected for relative UV response. The unusual degradant **33** has also been observed under some conditions, which reflects decarboxylation with chloride displacement, giving rise to a spiro-cyclopropyl group (Macherla, Mitchell, McArthur and Potts, unpublished observations).

Long-term and accelerated stability studies of **1** indicate that the API is highly stable when stored as a solid. The only observed degradation pathway involved exceedingly slow oxidation of the cyclohexene ring to the corresponding cyclohexenone **34** (Macherla, Manam and Potts, unpublished observation) (Scheme 1), the structure of which was confirmed by semi-synthesis (vide infra) and is reminiscent of the natural product salinosporamide C (**6**) (Figure 1) [51].

The above summary provides a window into the precautions required when generating and handling **1**, and serves as a preface to the following discussion on the opportunities and challenges of using **1** as a starting material for semi-synthesis.

## 4. Products of Semi-Synthesis

Process development for API manufacturing gave way to gram quantities of **1** to support preclinical studies and formulation development, and afforded an opportunity for the semi-synthesis of analogs. This windfall of material was tempered by challenges associated with the potential reactivity and instability of the functional groups of **1**, including the double bond of the cyclohexene ring, the β-lactone ring, and the chloroethyl group, as discussed above. Indeed, these same functional groups required careful consideration in the development of successful strategies for the total synthesis of **1** (vide infra). Nevertheless, we set out to modify the various structural elements using a classical semi-synthesis approach. With respect to the cyclohexenyl carbinol (P1 residue; Table 2), the C-5 secondary hydroxyl group proved difficult to derivative due to steric hindrance, as originally noted by Fenical and coworkers [15]. Oxidation to the ketone **35** [Dess-Martin periodinane; CH_2_Cl_2_] and subsequent reduction [NaBH_4_; monoglyme + 1% water; −78 °C] gave **36**, the C-5 epimer of **1** in 90% diastereomeric excess (*de*), along with parent compound **1** as a byproduct [34,60]. We subsequently explored many commercially available reagents and reaction conditions in attempt to control the *de* in favor of **1**, but without favorable outcome [60]. This led us to evaluate the potential for ketoreductase enzymes to execute the stereoselective reduction. After screening a library of ~100 ketoreductases, two enzymes (KRED-EXP-B1Y and KRED-EXP-C1A; BioCatalytics, Inc., Pasadena, CA) were identified that cleanly converted ketosalinosporamide **35** to **1** with complete stereoselectivity [60]. Foreknowledge of the utility of this reaction was strategic in the development of an endgame for our total synthesis of **1**, which was successfully completed upon executing the reduction as the final transformation in the sequence [41] (see Section 6.1.3. Nereus (Ling) Enantioselective Synthesis).

Efforts were also extended towards modification of the cyclohexene ring, the published scope of which encompassed reduction [10% Pd/C, H_2_; acetone] to give **18** [34], which was subsequently produced by mutasynthesis [64,65], epoxidation [mCPBA; CH_2_Cl_2_] of both faces of the cyclohexene ring (**37** and **38**) and subsequent halohydrin formation [HCl; acetonitrile] to give **39** [34]. Oxidation using *t*-BuOOH and CoAc_2_ produced a mixture of cyclohexenones, **40** and **34**; during reversed phase HPLC purification of the latter, intramolecular Michael addition of the lactam nitrogen to the cyclohexenone gave tetracyclic product **7** (Macherla, Manam and Potts, unpublished observations). We note that **7** is identical in structure to the hypothetical precursor to salinosporamide C (**6**) (Scheme 2) proposed by Williams *et al.* [51]. Oxidative cleavage of the cyclohexene ring double bond to create an acyclic substituent for further derivatization was also explored. In light of the role of P1 in recognizing the S1 specificity pocket of the proteasome substrate binding site, further exploration of P1 analogs is warranted. Efforts to generate P1 diversity using mutasynthesis are particularly encouraging [64,65] (see Section 2.4. Products of Mutasynthesis). In contrast, the only P1 analog generated to date by total synthesis is antiprotealide (**14**) [71,72], although this likely reflects the propensity of the synthetic organic chemistry community to target the parent natural product. Clearly, several key synthetic intermediates are excellent candidates for introducing novel P1 architecture (vide infra). With a variety of complementary methods now available, the authors anticipate a more extensive evaluation of P1, including the design and ‘synthesis’ (by any means) of subunit-specific inhibitors.

Semi-synthetic modifications to P2 (Scheme 3) were largely achieved through derivatization of two substrates, hydroxysalinosporamide (**41**) and iodosalinsoporamide (**42**). The parent (chlorinated) compound **1** was found to undergo a slow and low yielding transformation to iodosalinosporamide (**42**) [NaI; acetone; 11% in 6 days at RT]; bromosalinosporamide (**15**) was a superior starting material for this transformation [NaI; acetone; 84% in 2 days] but was not available in the same abundance as **1** in our laboratory [34,61]. The utility of **42** as a substrate for further analoging is clearly based on the greater propensity of iodide to displacement compared to chloride. Treatment of **42** with Gilman’s reagent in dry THF at −78 °C gave the corresponding propyl analog **9** [34], which we subsequently identified in *S. tropica* crude extracts as the natural product salinosporamide E [52]. Azido [NaN_3_; DMSO], propionate [Na(C_2_H_5_CO_2_); DMSO] and thiocyano [NaSCN, diethylamine; acetone] derivatives (**43**–**45**) were also prepared from **42** [34,59]. Despite the susceptibility of the β-lactone ring to base-catalyzed hydrolysis, brief treatment of **42** with NaOH [5N; acetone] afforded a complex mixture from which the first sample of hydroxysalinosporamide (**41**) was isolated, albeit in low yield [34]. Efforts to generate fluorosalinosporamide (**17**) to complete the halogen series using AgF in THF surprisingly gave the hydroxyl analog **41** as the major product (15%), with the target fluorinated **17** as a very minor byproduct [61]. AgF reagent has known utility in introducing fluorine in place of iodine or bromine [96,97], but in our hands gave rise to a hydroxy group. As **41** proved to be another important substrate for derivatization (vide infra), we sought to optimize its production by further exploring the utility of AgF reagents. This led to a 1-step method to convert the parent chlorinated natural product **1** directly to **41** using AgF supported on CaF_2_ in 35% yield (Macherla, Manam and Potts, unpublished observation). Hydroxyl analog **41** was subsequently used to generate analogs bearing non-halogen leaving groups, including mesyl, tosyl, and dansyl derivatives (**46**–**48**) [61], the latter of which may provide utility for fluorescence monitoring. Various carboxylate esters were also prepared. The P2 analog series has been evaluated for the ability to induce prolonged duration proteasome inhibition *in vitro* and firmly established the role of the leaving group at C-13 in inducing irreversible binding to the proteasome [61] (see 5. Structure-Activity Relationships).

With no evidence for naturally occurring thioesters in place of the β-lactone ring in *S. tropica* crude extracts (see Section 2.1. Natural Products of S. tropica), we endeavored to prepare them semi-synthetically. Treatment of **1** with methyl-3-mercapto-propionate or *N*-acetyl-L-cysteine methyl ester gave the corresponding thioesters **49** and **50**, which underwent slow and partial intramolecular nucleophilic displacement of chlorine to give cyclic ethers **51** and **52** (Figure 3). Salinosporamide B (**5**) was similarly derivatized (**53**). While proteasome inhibition assays suggested that the thioester may directly react with the proteasome, those species that retained the potential to reform the β-lactone ring *i.e.*, **49**, **50** and **53**, were more potent inhibitors of proteasome activity than cyclic ethers **51** and **52** [52] (see Section 5. Structure-Activity Relationships). As premature chloride elimination disables the molecule’s full inhibitory potential, it is no wonder that thioester analogs of the salinosporamides have not been found in nature. Nevertheless, if the cellular metabolism pathways identified for omuralide and lactacystin [27,98] are relevant to the salinosporamides, then thioesters may indeed be generated upon *in vivo* administration of **1**. Preliminary studies suggest that this may be the case.

## 5. Structure-Activity Relationships

Structure-activity relationship (SAR) trends are evaluated below with respect to inhibition of CT-L activity against purified 20S proteasomes. IC_50_ values for P1 and P2 analogs are captured in Tables 2 and 1, respectively.

### 5.1. β-Lactone Derivatives

The β-lactone ring of **1** and other salinosporamides directly acylates the proteasome active site residue Thr1O^γ^ (Figure 2), as demonstrated by crystal structures of various analogs in complex with the yeast 20S proteasome [35,67]. It is therefore not surprising that modification of the β-lactone ring has a major impact on proteasome inhibition. Indeed, degradation product **28** (Scheme 1) shows no CT-L inhibitory activity at the highest concentrations tested (IC_50_ > 20 μM) [34]; this is consistent with the loss of both the β-lactone that acylates the proteasome and the chloroethyl trigger that induces sustained proteasome inhibition (vide infra). It was not possible to directly evaluate β-lactone hydrolysis product **27** due to rapid conversion to **28** upon attempted purification. While thioesters of the salinosporamides are not found in nature, they have been generated by semi-synthesis (Figure 3) [52]. The thioester derivative of salinosporamide B is only half as potent as its β-lactone precursor (**5**: IC_50_ = 26 nM; **53**: IC_50_ = 50 nM). Interestingly, when salinosporamide A (**1**) was similarly derivatized, the corresponding thioester could be isolated in both the C-3OH (*seco*) (e.g., **49**, **50**) and cyclic ether (e.g., **51**, **52**) forms. The thioester derivative in the cyclic ether form retains modest proteasome inhibitory activity (**51**: IC_50_ = 230 nM), indicating that the less reactive thioester can still bind and inhibit CT-L activity, but with ~100-fold less potency than **1** (IC_50_ = 2.5 nM). In the *seco* form, C-3O can either displace chloride or undergo the competing reaction of *in situ* β-lactone reformation. Since the *seco* form is ~25-fold more active (**49**: IC_50_ = 9.3 nM) than the corresponding cyclic ether **51**, but only ~4-fold less potent than the β-lactone **1**, the dominant pathway appears to be reformation of the β-lactone ring to give the highly activated species [52]. This follows the precedent of lactacystin, which similarly gives omuralide in cells [26,27,98].

### 5.2. P1 Analogs

Natural products chemistry and semi-synthesis provided an opportunity to evaluate the role of C-5OH. Salinosporamide J (**13;** C-5H_2_) is a 20-fold less potent inhibitor of CT-L activity (IC_50_ = 52 nM) than the parent **1**, but significantly more active than ketosalinosporamide (**35**) and C-5-*epi*-salinosporamide (**36**) (IC_50_ = 8.2 μM and >20 μM, respectively). Thus, reduction of the C-5 hydroxyl group to a methylene group is preferred to epimerization or oxidation [34,52]. The crystal structure of **1** in complex with the yeast 20S proteasome revealed hydrogen-bonding interactions between the ligand C-5OH and the proteasome Thr21NH, and further suggested that **35** and **36** may introduce steric interactions that are not well tolerated [35]. In the case of **13**, the hydrogen bonding potential is lost, but problematic steric interactions would not be expected, in agreement with the trends in the assay data [IC_50_ **1** < **13** (C-5H_2_), **35** (keto-C-5) < **35** (epi-C-5OH)] [34,35,52].

Cyclohexene ring modification or replacement has been achieved by mutasynthesis [64,65], semi-synthesis [34] and total synthesis [71,72]. The P1 analogs generated to date largely represent hydrophobic hydrocarbons, for which the potency rank order is cycloalkenyl > cycloalkyl > branched aliphatic > linear aliphatic > aromatic, with respect to inhibition of CT-L activity. The reduced potency of the phenyl analog is in agreement with SAR studies of omuralide [29]. Epoxidation of the cyclohexene ring is only well tolerated on one face (**37**: IC_50_ = 6.3 nM versus **38**: IC_50_ = 91 nM) [34]. Ring contraction from a 6- to a 5-membered ring gave promising results; the cyclopentenyl analog is ~equipotent with **1** with respect to inhibition of CT-L activity and more cytotoxic against human colon carcinoma HCT-116 cells [65]. Given the role of P1 in recognizing the S1 specificity pocket of the proteasome, which largely imparts the CT-L, T-L and C-L sites with their substrate cleavage preferences, the authors stress the importance of evaluating P1 analogs against all three proteolytic sites, which may reveal unique inhibition profiles across subunits.

### 5.3. P2 Analogs

Fermentation extracts of *S. tropica* contained low levels of compounds **10**, **16**, and **11**, the C-2 epimers of **1**, **5**, and **8** [34,52]. These 2(*S*) diasteromers were >50-fold less potent than their 2(*R*) congeners with respect to inhibition of CT-L activity, indicating that the 2(*R*) stereochemistry of the major secondary metabolites is well optimized. In the case of **1**, this stereoconfiguration is particularly important: the *syn* relationship of the chloroethyl and C-3OH substituents of the γ-lactam ring supports intramolecular nucleophilic displacement of chloride to give a *cis*-fused bicyclic lactam upon binding to the proteasome (Figure 2), which results in irreversible proteasome inhibition *in vitro* [35,61].

P2 analogs were highly instrumental in establishing the mechanistic role of the chloride leaving group. Crystallographic studies and proteasome inhibition/recovery experiments using purified 20S proteasomes confirmed that displacement of chloride (or alternative leaving groups) at the proteasome active site results in irreversible inhibition of proteasome activity [35,61,67]. The irreversible inhibitors, which bear a leaving group at the C-13 position of P2 [e.g., Cl (**1**), Br (**15**), I (**42**), and various sulfonate esters (**46**, **47**, **48**)] are generally more potent proteasome inhibitors when compared to their slowly reversible congeners, which do not bear a leaving group at this position [61]. Interestingly, fluorosalinosporamide (**17**) behaves intermediately, which is attributed to the poor leaving group potential of fluoride [61]. Slow fluoride elimination in the proteasome active site was nicely captured in freeze-frame (short and long soak) crystal structures of **17** in complex with yeast 20S proteasomes [67]. These findings are in good agreement with its behavior as a partially reversible proteasome inhibitor [61] and its intermediate behavior between **1** and **5** [61,63]. However, P2 analogs that do not bear a leaving group are still very potent inhibitors of purified 20S proteasomes, with IC_50_ values in the low nM range (Table 2). Thus, while the kinetic distinction between slowly reversible and irreversible inhibition of purified proteasomes is evident, the greatest impact of irreversible binding is on cellular events downstream of proteasome inhibition, whereby sustained proteasome inhibition leads to potent cytotoxicity in tumor cells. Indeed, P2 analogs bearing a leaving group exhibit much more potent cytotoxicity in hematological and solid tumor cell lines [34,51,59]. A comprehensive discussion of this and other lessons learned from β-lactone proteasome inhibitors is currently in review (M. Groll and B. Potts, 2010).

## 6. Total Synthesis of 1

In this section, the total synthesis of **1** is reviewed. At the time of writing, 5 enantioselective total syntheses [39–43], 2 racemic syntheses [44,45], and 5 formal syntheses [46–50] have been published, reflecting a wide variety of strategies that often converge to common advanced intermediates. The strategies are captured in Schemes 4–15, in which the atom numbering for all intermediates (including well known starting materials or their derivatives) correlates with the atom numbers of the final synthetic target **1** (Figure 1). The reader is directed to the source articles for supporting references.

### 6.1. Enantioselective Synthesis of (-)-**1**

#### 6.1.1. Corey Enantioselective Synthesis

The first total synthesis of **1** was reported by Corey and coworkers [39], marking a key milestone and setting the standard for all who followed. Key features of this innovative route included: (i) an intramolecular Baylis-Hillman aldol reaction to construct the γ-lactam with the desired stereochemistry at the C-3 tertiary alcohol; and (ii) simultaneous construction of the C-5/C-6 stereocenters by allylation of a late-stage intermediate aldehyde with 2-cyclohexenyl zinc chloride.

The overall synthesis is captured in Scheme 4. (*S*)-threonine methyl ester served as a natural choice for the starting material, comprising the C-15/C-4/C-3/C-14 contiguous carbons of **1**. *N*-acylation with 4-methoxybenzoyl chloride and subsequent *p*-TsOH catalyzed cyclization gave the corresponding oxazoline **1-2**. Stereoselective alkylation with ClCH_2_OBn afforded **1-3** with the desired chirality at the quaternary C-4 stereocenter while effectively introducing C-5. Reductive oxazoline ring opening with NaBH_3_CN-HOAc gave PMB derivative **1-4**. After TMS-protection, selective *N*-acylation with acrylyl chloride and acidic workup installed the contiguous 3-carbon unit C-1/C-2/C-12; subsequent Dess-Martin periodinane oxidation afforded keto amide ester **1-5** in preparation for γ-lactam formation in the next step. This was initially achieved *via* a quinuclidine base-catalyzed intramolecular Baylis-Hillman aldol reaction that occurred over 7 days to give γ-lactam **1-7** with the desired C-3 stereochemistry with high selectivity (9:1). Both the efficiency and stereoselectivity of this important step were subsequently improved with an alternative cyclization strategy that was reported independently (vide infra) [72]. The corresponding silyl ether underwent tri-*n*-butyltin hydride mediated radical cyclization to the *cis*-fused γ-lactam **1-8**. The benzyl ether was cleaved and the resulting alcohol oxidized to obtain key intermediate aldehyde **1-9**, which was reacted with 2-cyclohexenyl zinc chloride to complete the construction of the P1 cyclohexenyl carbinol residue. Notably, this diastereoselective allylation introduced the contiguous C-5/C-6 stereocenters simultaneously and in high stereoselectivity (20:1). Testament to the remarkable utility of this step is best offered by the synthetic routes that subsequently adopted it (vide infra). Tamao-Fleming oxidation of **1-10** followed by deprotection of the lactam nitrogen gave triol **1-11.** Finally, the methyl ester was hydrolyzed to set the stage for clean and efficient β-lactone formation and chlorination in one pot to give (-)-**1** for the first time by total synthesis.

This “simple stereocontrolled synthesis” of **1** by Corey and coworkers [39] was subsequently improved in overall efficiency by replacing the Baylis-Hillman reaction (7 days) with a diastereoselective cyclization sequence; treatment of **1-5** with Kulinkovich reagent followed by iodination and HI elimination was completed over 5 hours (overall sequence) with remarkable selectivity (*dr* > 99:1). The resulting, highly functionalized γ-lactam **1-7** is a versatile intermediate, serving as a common precursor to **1** and hybrid analogs antiprotealide (**14**), β-methyl omuralide, and other potential analogs [72]. This intermediate was later targeted in the formal synthesis of **1** by Langlois and coworkers (vide infra) [46]. Moreover, a precursor related to **1-5** but comprising the isopropyl carbinol P1 moiety was also advanced to **14** [71], further demonstrating the utility of this transformation.

#### 6.1.2. Danishefsky Enantioselective Synthesis

The enantioselective synthesis of (-)-**1** from a bicyclic derivative of L-glutamic acid was reported by Endo and Danishefsky in 2005 [40]. This novel synthesis features a cationic hemiacetal-mediated phenylselenenylation of an exocyclic methylene to stereoselectively install the quaternary center at C-3; this step, together with subsequent radical deselenylation to provide the C-3 methyl substituent, were later adopted by Hatakeyama and coworkers’ in their formal synthesis of **1** [42].

The total synthesis, captured in Scheme 5, exploited the strong facial bias of pyroglutamate derivative **2**-**1** to attack at C-3 from its β-face, with subsequent alkylation at C-2 from its α-face. The vinyl group of the C-3 substituent of intermediate **2**-**2** was advanced to a carbonate ester acylating agent for subsequent intramolecular and stereoselective delivery to C-4. This required that the lactam functionality be masked in the form of the imidate ester **2**-**4** to enable exclusive anion formation at C-4. The resulting lactone **2**-**5** carried an advanced stereochemical imprint for further evolution to **1**. Nucleophilic ring opening of the lactone was achieved regioselectively with a phenylselenium ion, and the resulting carboxylic acid was benzylated to give **2**-**6**, thereby differentiating C-5 and C-15. The C-3 and C-2 substituents were then converted to exocyclic methylene and acetaldehyde moieties, respectively (**2**–**7**). This set the stage for the hemiacetal-mediated phenylselenenylation of the exocyclic methylene, which gave **2-8**, thereby establishing the C-3 quaternary center with complete stereocontrol. Subsequent radical deselenylation of **2-8** provided the C-3 methyl substituent. With the C-2/C-3/C-4 contiguous stereocenters in place, the benzyl ester was converted to the corresponding C-5 aldehyde **2**-**9**. Introduction of the cyclohexenyl group was achieved by adopting the elegant method established by Corey and coworkers (vide supra) [39]; indeed, allylation using cyclohexenyl zinc chloride occurred with the desired stereochemical outcome at C-5 and C-6 to give **2**-**10**. The authors noted that allylation of the corresponding imidate aldehyde substrate (derived from **2**-**4**) gave poor diastereoselectivity with the same reagent, highlighting the importance of the PMB protecting group for diastereoselection [40]. Finally, the corresponding triol **2**-**11** was unveiled for β-lactone formation and replacement of the primary alcohol with chloride, *ala* Corey and coworkers [39].

#### 6.1.3. Nereus (Ling) Enantioselective Synthesis

In our own laboratory, a novel enantioselective strategy was envisioned by Taotao Ling that involved an intramolecular aldol cyclization to generate key intermediate **3-4** using the Self-Regeneration of Stereocenters (SRS) principle developed by Seebach *et al.* [99], as captured in Scheme 6 [41]. The advantage of this approach was the efficient, scalable, and simultaneous generation of three contiguous stereocenters C-2/C-3/C-4, in contrast with earlier syntheses that employed their stepwise introduction [39,40].

Enantiomerically pure oxazolidine-γ-lactam **3-4** was prepared from β-keto amide **3-3**, where the C-4 chirality (derived from D-serine) was maintained during the intramolecular aldol cyclization following a strategy previously described by Andrews *et al.* [100] and the C-2 and C-3 stereocenters were simultaneously constructed in a substrate-directed fashion. The resulting, highly functionalized intermediate **3-4** served as a key precursor for the enantioselective total synthesis of (-)-**1**. Thus, the D-serine derived oxazolidine served as both a chiral directing group during the intramolecular aldol cyclization and as a protecting group during subsequent steps of the synthesis, and would ultimately be unveiled to allow oxidation of C-15 in anticipation of β-lactone formation in the last stages of the synthesis. Compound **3-4** was advanced to aldehyde **3-6** in preparation for allylation. Cyclohexene ring installation using Corey’s method (with cyclohexenyl zinc chloride) [39] indeed gave an *anti* addition product, but with both undesired C-5 and C-6 stereocenters. This clearly distinguished our oxazolidine protected substrate **3-6** from the PMB-protected γ-lactam used in other routes [39,40,44]. We therefore turned to Brown’s allylboration chemistry (*i.e.*, coupling of **3-6** with *B*-2-cyclohexen-1-yl-9-BBN [101]), which was expected to give a *syn* addition product. Fortunately, the product **3-7** had the desired stereochemistry at C-6; thus, the required stereochemistry at C-5 would need to be generated later, which was known to be feasible based on our prior development of selective semi-synthetic transformations on the natural product [60] (see 4. Products of Semi-Synthesis). With the overall carbon skeleton in place, the oxazolidine-protected alcohol (C-15) was revealed (**3-9**) and oxidized in preparation for β-lactone formation, followed by halogenation of the C-2 sidechain to give **3-11** (equivalent to compound **36**), the C-5 epimer of **1**. The final C-5 stereocenter was established by Dess-Martin periodinane oxidation to the corresponding ketone **3-12** (equivalent to compound **35**), which was stereoselectively reduced by a ketoreductase enzyme [41,60] to afford (-)-**1**.

In summary, the key features of the enantioselective route developed in our laboratory included intramolecular aldol cyclization to simultaneously generate the three contiguous stereocenters of intermediate **3**-**4**, of which 100g of material was produced *via* this scaleable process; cyclohexene ring addition using *B*-2-cyclohexen-1-yl-9-BBN; and inversion of the C-5 stereocenter by oxidation followed by enantioselective enzymatic reduction.

#### 6.1.4. Hatakeyama Enantioselective Synthesis

Hatakeyama and coworkers’ total synthesis of (-)-**1** (Scheme 7) [42] represents a successful application of the construction of highly functionalized pyrrolidinones using an indium-catalyzed Conia-ene reaction. Conia-ene reactions [102] generally require harsh conditions, under which racemization and isomerization of the exocyclic olefin from the β,γ - to the α, β-position are of considerable concern, while metal catalyzed reactions may be carried out under milder conditions. If the target pyrrolidinone **4-5** could be obtained by this strategy, advancement to (-)-**1** was envisioned as follows. The C-3 quaternary center would be constructed stereoselectively by intramolecular delivery of oxygen from the C-2 substituent to the exo olefin, as established by Endo and Danishefsky [40], while the C-4 center could be created by selective reduction of one of the geminal esters of the resulting bicyclic intermediate. This would set the stage for cyclohexenyl zinc chloride addition, per Corey and coworkers [39].

The synthesis was executed as outlined above. Specifically, to prepare amide **4-4**, the substrate for the key In(OTf)_3_-catalyzed cyclization, chiral propargyl alcohol **4-1** was converted to the mesylate, which was then reacted with (*t*-butyldimethylsilyloxy) acetaldehyde *via* the allenylzinc species to give **4-2** as a 9:1 epimeric mixture. Removal of the PMB group, selective acetylation, and desilylation afforded **4-3**, which was treated with CrO_3_ and HIO_4_ in aqueous acetone to obtain the corresponding carboxylic acid. Subsequent condensation with dimethyl-2-(4-methoxybenzylamino)malonate *via* the acid chloride afforded the key precursor **4**-**4,** anticipating Conia-ene cyclization. Interestingly, during purification on silica gel, amide **4-4** partially underwent cyclization to give an inseparable mixture of **4-4** and **4-5** (72:28) [Further subjecting this mixture to silica gel chromatography conditions gave **4-5** quantitatively and in 90% *ee*, suggesting a silica gel promoted Conia-ene reaction (rather than cyclization through the corresponding achiral allenylamide) [42,103]]. Treatment of the mixture of **4-4** and **4-5** with a catalytic amount of In(OTf)_3_ in toluene at reflux (the original conditions developed for the Conia-ene cyclization!) indeed resulted in complete conversion of **4-4** into **4-5**. Importantly, no significant loss of enantiomeric purity was observed. Having demonstrated this key transformation, the acetoxy group of **4-5** (a base labile intermediate) was hydrolyzed under mild lipase-catalyzed reaction conditions to give the corresponding alcohol, which was then oxidized to aldehyde **4-6**. This set the stage for the assembly of the C-3 quarternary center, which was achieved according to the precedent established by Endo and Danishefsky [40] to obtain **4-7** (vide supra). Radical deselenenylation of **4-7** was followed by selective NaBH_4_ reduction, which nicely discriminated between the geminal esters, after which Dess-Martin periodinane oxidation afforded aldehyde **4-8**. Reaction of **4-8** with cyclohex-2-enylzinc chloride according to Corey and coworkers [39] yielded **4-9** as a single stereoisomer. Removal of the PMB group followed by reductive ring opening of the cyclic acetal afforded known triol **4-10** (*i.e.*, identical to **1**-**11**, Scheme 4). Finally, dealkylative cleavage of the methyl ester was promoted by (Me_2_AlTeMe)_2_, adopted from Mulholland *et al.* [44] (vide infra), followed by β-lactonization and chlorination to obtain (-)-**1**.

#### 6.1.5. Omura Enantioselective Synthesis

It is most fitting that Omura and coworkers, who first discovered lactacystin (**2**) [23,24], have developed a total synthesis of (-)-**1** [43]. Their novel strategy (Scheme 8) presents with the early construction of the cyclohexene ring, with introduction of the C-5/C-6 stereocenters *via* a chelation-controlled aldol reaction. This represents a distinct approach from the many routes that adopted the Corey strategy [39] to install the cyclohexene ring. The Omura synthesis also features an intramolecular aldol reaction to construct the lactam C-2/C-3 bond and an intermolecular Reformatsky-type reaction followed by 1,4-reduction to generate the P2 substituent.

The initial phase of the total synthesis of (-)-**1** comprised generation of aldehyde **5**-**4** in preparation for cyclohexanone addition, and subsequent cyclohexene formation. Towards this end, optically active acetate **5**-**2** was prepared by Wittig olefination of aldehyde **5-1**, followed by hydrolysis to the corresponding diol, enzymatic desymmetrization, and TBDPS protection of the remaining primary alcohol. Then, the corresponding MEM ether underwent intramolecular cyclic carbamation and *N*-PMB protection to obtain **5-3**, which was subjected to osmium-catalyzed dihydroxylation followed by oxidative cleavage of the corresponding diol to give aldehyde **5-4**. This set the stage for the addition of cyclohexanone *via* a chelation-controlled aldol reaction quenched with BzCl that effectively installed both of the desired C-5 and C-6 stereogenic centers of intermediate **5-5**. The next step was the conversion of cyclohexanone to cyclohexene, a challenging problem that was solved by stereoselectively generating the *anti* 1,3-diol and derivatization to the corresponding cyclic sulfate **5-6**, the elimination of which occurred in high yield to give the desired cyclohexene **5-7**.

The next phase of the synthesis involved construction of the γ-lactam ring and the quaternary C-3 stereocenter. Intramolecular transcarbamation of **5-7** with NaH followed by Swern oxidation gave aldehyde **5**–**8**, which was advanced to the corresponding ketone to provide the required methyl group of **1**. The nitrogen was deprotected to give **5**-**9**, enabling construction of the γ-lactam and C-3 stereogenic center *via N*-acylation followed by an intramolecular aldol reaction using LHMDS and chloroacetyl chloride in one pot, which generated the desired γ-lactam **5-10** as a single isomer.

In the final stages of the synthesis, the C-2 side chain (P2) was installed using a SmI_2_-mediated intermolecular Reformatsky-type reaction of **5-10** with benzyloxy acetaldehyde. The resulting β-hydroxy-γ-lactam **5-11** was converted to the α,β-unsaturated lactam by mesylation-elimination, followed by alkaline hydrolysis and stereoselective 1,4-reduction with LiEt_3_BH to obtain **5-12**. A series of selective protection and deprotection steps afforded **5-13**, in preparation for oxidation of the primary alcohol to enable β-lactonization and finally, chlorination, to generate the desired β-lactone **5-14**, which was deprotected to afford (-)-**1**.

### 6.2. Racemic Synthesis of (±)-**1**

#### 6.2.1. Pattenden Racemic Synthesis

The concise racemic route developed by Pattenden and coworkers (Scheme 9) was first communicated in 2006 [44], and a full account was reported in 2008 [104]. The straightforward 14-step total synthesis commenced with intramolecular aldol cyclization of protected β-keto-amide **6**-**2** to generate γ-lactam (±)**-6**-**3**. This approach nicely parallels the strategy pursued in our own laboratory [41], albeit without enantioselective control. Nevertheless, the cyclization gave the required relative stereochemistry at C-2 and C-3, which was controlled with careful attention to the temperature of this deprotection-aldol cyclization reaction. After TMS and PMB protection of the tertiary alcohol and lactam nitrogen, respectively, dimethyl ester **6**-**4** underwent regioselective superhydride reduction to give C-5 aldehyde **6**-**5**; specifically, the methoxycarbonyl *trans* to the sterically hindered C-3OTMS group of intermediate **6**-**4** underwent selective reduction, successfully exploiting the facial bias of the substrate. The remainder of the synthesis, including stereoselective allylation with cyclohexenyl zinc bromide, was concluded in analogy to the strategy of Corey and coworkers [39] to give (±)**-1**. Of note, dimethylaluminium methyltelluride (60% yield) was used in place of 3M LiOH (<10% yield) to hydrolyze the methyl ester of **6**-**7**, prior to lactonization and chlorination.

#### 6.2.2. Romo Racemic Synthesis

The synthesis of (±)-**1** by Romo and coworkers [45] was a natural extension of their keen interest in constructing carbocycle-fused β-lactones (e.g., [105]). Their strategy comprised the coupling of an α-amino acid with a heteroketene dimer, the product of which underwent nucleophile promoted bis-cyclization to simultaneously construct the highly functionalized β-lactone-γ-lactam core (±)-**7-6a**. The cyclohexenyl group was successfully introduced at the penultimate stage of the synthesis, demonstrating the stability of both the β-lactone and chloroethyl functionalities to the conditions of this key reaction, thereby suggesting strong potential for the generation of a variety of P1 analogs from late stage aldehyde intermediate **7-7**.

The details of this concise synthetic route are captured in Scheme 10. *N*-PMB serine allyl ester **7-2** was coupled with heteroketene dimer **7-3**; this key interemediate was reportedly readily generated in gram quantities. The resulting β-keto amide underwent Pd-mediated ester deprotection to give **7-4** in anticipation of the key bis-cyclization reaction, which was executed with modified Mukaiyama reagent **7-5** to activate the carboxylic acid in the form of a pyridone ester (not shown). The desired β-lactone-γ-lactam **7-6a** was obtained in 2-3:1 *dr*. Deprotection of the benzyl ether followed by modified Moffatt oxidation to the corresponding aldehyde **7-7** set the stage for treatment with cyclohexenyl zinc chloride to complete the P1 moiety and simultaneously establish the C-5/C-6 stereocenters, according to the precedent of Corey and coworkers [39]. Final unveiling of the lactam nitrogen gave (±)-**1**. This approach was also extended to the total synthesis of the related (±)-cinnabaramide A [45].

### 6.3. Formal Synthesis of **1**

#### 6.3.1. Langlois Formal Synthesis

Langlois and co-workers [46] developed a synthesis of (2*R*,3*S*)-α-methylene-γ-lactam **8-10**, a key intermediate in Corey and coworkers’ synthesis of **1** [39]. This formal synthesis represents an extension of their earlier efforts in which the corresponding *N*-Boc racemic scaffold was generated [106], providing a foundation for the development of an asymmetric route to *N*-PMB intermediate **8-10** from (*S*)-methyl 2-hydroxymethyl pyroglutamate (**8-2**) [46].

The formal synthesis of **1** is outlined in Scheme 11. Chiral intermediate (*S*)-**8-2** had been previously generated by Langlois and Nguyen from bicyclic nitrile **8-1** in their synthesis of deoxydysibetaine [107] and was prepared accordingly. Then, selective *O*-benzylation of (*S*)-**8-2** was achieved with 2-benzyloxy-1-methylpyridinium triflate as a mild and nearly neutral benzylating agent in the presence of MgO, giving (*S*)-**8-3** in 75% yield. According to their prior work [106], the subsequent steps in the synthesis would not induce racemization, thus, the remainder of the formal synthesis was demonstrated using racemic **8-3**. Towards this end, the *N*-PMB derivative of **8-3** was generated (not shown), however, subsequent introduction of the conjugate double bond was low yielding compared to that achieved previously with the corresponding *N*-Boc derivative. Consequently, introduction of the PMB group was reserved for a later stage of the synthesis, and **8-3** was instead Boc-protected for advancement to **8-6** by means established previously [106]. Specifically, introduction of the conjugate double bond was achieved *via* phenylselenylation using LDA as a base followed by selenoxide elimination; the resulting intermediate **8-4** could be used to prepare β-methyl unsaturated lactam **8-6** *via* two possible pathways: (*i*) treatment with diazomethane to give pyrazolines **8-5a** and **8-5b**, followed by thermolysis (overall 35%); or (*ii*) stereoselective addition of the C-3 methyl group using methylcuprate to give **8-5c**, with subsequent introduction of the double bond *via* phenylselenylation and selenoxide elimination (overall 63%). Thereafter, the Boc group of pyrrolinone **8-6** was removed and the nitrogen was PMB-protected to give **8-7**. This set the stage for the selective 1,3-dipolar cycloaddition of *N*-methylnitrone to simultaneously introduce the C-3 oxygen and the precursor to the exo-methylene group. Specifically, formation of cycloadducts **8-8a** and **8-8b** was achieved by heating **8-7** with *N*-methylnitrone in toluene, which gave **8-8a** as the major product. The isoxazolidine ring was hydrogenolyzed in the presence of Pd(OH)_2_, affording **8-9**. Finally, the target α-methylene-γ-lactam **8-10** was obtained by forming the trimethylammonium salt with iodomethane in methanol, which was further treated with a biphasic mixture of aqueous Na_2_CO_3_ and CH_2_Cl_2_ (4 days at room temperature) to induce elimination to the exo-methylene group in high yield [46]. This completed the formal synthesis, as intermediate **8-10** was previously advanced to **1** by Corey and coworkers [39].

#### 6.3.2. Lam Formal Synthesis

Lam and co-workers achieved a formal synthesis of **1** using a sequential nickel-catalyzed reductive aldol cyclization-lactonization reaction as a key step [47]. α,β-unsaturated amide **9-3** was targeted as a highly functionalized substrate for this reaction, which would give rise to an advanced γ-lactam comprising the precursor to the P2 substituent. A related cyclization of a less densely functionalized substrate had been achieved previously [108]. Thus, application to **9-3**, which was more sterically congested and comprised several Lewis basic groups that could potentially bind the catalyst and reductant and divert the course of the intended reaction, along with only a single stereocenter to control the absolute configurations of the two new centers generated upon cyclization, presented significant challenges that would rigorously test this methodology.

The formal synthesis is captured in Scheme 12 and commenced with Swern oxidation of known amino alcohol **9-1** [39] to give aminoketone **9-2**, which was then suitably acylated to afford the target α,β-unsaturated amide **9-3** in high yield. This set the stage for the key reductive aldol cyclization reaction, which was attempted using a variety of conditions. Commercially available nickel-phosphine complexes (Ph_3_P)_2_NiBr_2_ and (Me_3_P)_2_NiCl_2_ were identified as effective precatalysts when used in conjunction with Et_2_Zn reductant, and although the target **9-4c** was not formed, a fused γ-lactam-γ-lactone **9-5a** (presumably generated *via* **9-4a**) with the desired stereochemistry was isolated in 35% and 42% yields, respectively. This provided the unexpected benefit of protecting the C-3 tertiary alcohol during subsequent steps. To complete the synthesis, **9-5a** underwent Pd-catalyzed debenzylation to afford alcohol **9-6**, which was oxidized to the corresponding aldehyde **9-7** *via* Dess-Martin periodinane oxidation [47]. Due to its relative instability, the aldehyde was immediately reacted with 2-cyclohexenylzinc chloride as described by Corey and coworkers [39] to give homoallylic alcohol **9-8**. The formal synthesis was completed by reductive ring opening of the **9-8** lactone using NaBH_4_ to give triol **9-9**, for which the conversion to **1** had been previously described by the Corey [39] and Pattenden groups [44].

#### 6.3.3. Bode Formal Synthesis

Struble and Bode have explored *N*-heterocyclic carbene (NHC) catalyzed intramolecular lactonization to prepare densely functionalized bicyclic γ-lactam-γ-lactone adducts from enals. This methodology was applied to their formal synthesis of **1** (Scheme 13) based on its potential for a concise and high yielding entry to the core bicyclic γ-lactam-γ-lactone scaffold **10-6a** [48].

The synthesis of **10-1** from L-threonine was established previously by Corey and coworkers in their synthesis of **1** [39]. To generate target aldehyde **10-4,** compound **10-1** was derivatized to **10-2** using a three step sequence comprising *N*-acylation with sorbyl chloride, silyl ether cleavage under aqueous acidic conditions and Dess-Martin oxidation. Subsequent regioselective sharpless dihydroxylation at the γ,δ-position of **10-2** afforded diol **10-3** as a single diastereomer. The diol was cleaved with sodium periodate to yield terminal aldehyde **10-4**, setting the stage for the key NHC-catalyzed asymmetric intramolecular cyclization. Using *N*-mesityl aminoindanol derived chiral triazolium catalyst **10-5**, bicyclic γ-lactam-γ-lactone diastereomers **10-6a** (desired) and **10-6b** (undesired) were obtained in high yield (88%) in a diastereomeric ratio of 1:1.1. γ-Lactam-γ-lactone **10-6a** is identical to **9-5a** (Scheme 12) targeted by Lam and coworkers [47], thereby representing a formal synthesis of **1**.

#### 6.3.4. Tepe Formal Synthesis

Mosey and Tepe demonstrated the utility of an ene-type alkylation reaction to construct Corey’s intermediate ketone **11-7** [39] using an oxazol-5-(4*H*)-one (“oxazolone”) and an enol ether in their formal synthesis of (±)-**1** (Scheme 14) [49]. The broader utility of oxazolones in the construction of other natural products has also been reviewed by Tepe and coworkers [109].

Ene-type alkylation of racemic oxazolone **11-1** with *t*-butyl enol ether followed by sodium borohydride reduction gave **11-2** in 88% yield as a 3:1 mixture of diastereomers. The major diastereomer was advanced to the target, however, the authors observe that both diastereomers could be used to generate (±)-**1**, since the second stereocenter (C-3) is ultimately destroyed during oxidation of **11-6** or **11-9** to the corresponding ketone **11-7**. The amide functionality of **11-2** was selectively reduced by dehydrative cyclization under basic conditions with MsCl, followed by reduction of the resulting oxazoline **11-3** with NaCNBH_3_ in acetic acid to afford *N*-PMB-protected amino alcohol **11-4**. The primary alcohol was subsequently protected to give benzyl ether **11-5**. Acylation with acrylyl chloride was smoothly executed according to Corey and coworkers [39] to obtain amide **11-8**. However, deprotection of the *t*-butyl group with TFA or phosphoric acid to obtain alcohol **11-9** as a single diastereomer was low yielding (25% or 27%), suggesting that earlier stage removal may be preferable. Indeed, conversion of **11-5** to **11-6** was more rapid (6h) and high yielding (98%). Target ketone **11-7** could be obtained from either **11-6** or **11-9** according to Corey’s synthesis [39], thereby completing the formal synthesis of (±)-**1**.

#### 6.3.5. Chida Formal Synthesis

Chida and coworkers previously demonstrated that Overman rearrangement on sugar-derived scaffolds followed by further exploitation of residual carbohydrate functional groups is a successful methodology for the synthesis of natural products comprising α-substituted α-amino acids, including lactacystin [110]. Accordingly, Momose *et al.* reported the formal synthesis of (-)-**1** from D-glucose, featuring Overman rearrangement of allylic trichloroacetimidate **12-14** to construct the quaternary C-4 stereocenter bearing nitrogen (**12-15**). During this step, the chiral information was relayed from the C-3 stereocenter, which had been previously generated with complete stereoselectivity under substrate control by reaction of D-glucose-derived cyclic ketone **12-8** with Me_3_Al (Scheme 15) [50].

The synthesis commenced with diacetone-D-glucose, which was advanced to primary alcohol **12-1** in 4 steps according to Fleet *et al.* [111], followed by protection with BnBr to obtain **12-2**. The exocyclic acetonide was selectively cleaved to the corresponding diol **12-3**, which was suitably protected (**12-5**). Hydrolysis of the remaining acetonide and cleavage of the resulting glycol afforded pyranose derivative **12-6**. The corresponding PMB β-glycoside was generated and the *O*-formyl group removed to give pyranoside **12-7**, which was oxidized with DMSO-Ac_2_O to obtain ketone **12-8** in preparation for generation of the C-3 tertiary alcohol. This important step was achieved stereoselectively upon reaction with Me_3_Al in toluene, which afforded **12-9** as the sole product in high yield. The high stereoselectivity occurred under substrate control, specifically, in the presence of the bulky alkyl side chain and the OTs group flanking the ketone carbonyl. Tosyl group deprotection was achieved using Mg in MeOH to give **12-10**, followed by oxidation of the secondary alcohol to a ketone (**12-11**) in anticipation of generating the key allylic trichloroacetimidate **12-14**. Towards, this end, **12-11** underwent Horner-Wadsworth-Emmons reaction with subsequent TMS protection of the tertiary alcohol to afford *E*-alkene **12-12** as a single isomer. After reduction of the ester with DIBAL-H, the primary alcohol **12-13** was converted into trichloroacetimidate **12-14** *via* CCl_3_CN and DBU. This set the stage for the centerpiece of the synthesis, the Overman rearrangement, which was executed in *t*-butyl benzene at 150 °C in the presence of Na_2_CO_3_ in a sealed tube for 2 days, and gave rise to the desired isomer **12-15** and its C-4 epimer (not shown) in 69% and 16% isolated yields, respectively. The observed stereoselectivity was rationalized by considering both steric and electronic factors of two possible chair-like transition state intermediates, with the desired isomer obtained from the transition model which did not give rise to repulsive interactions between the nitrogen and the neighboring TMS group.

Having successfully demonstrated the key transformation, **12-15** was advanced to Corey’s intermediate **12-24** [39]. First, the acetal needed to be transformed to a hemiaminal that could be oxidized to the desired γ-lactam. Towards this end, the trichloroacetyl group in **12-15** was replaced with Cbz to give **12-16**. Then, hemiaminal formation was achieved in two ways: i) removal of the PMB group with DDQ and subsequent treatment with TBAF revealed hemiacetal **12-17**, which was spontaneously converted into 5-membered hemiaminal **12-18** (67%); and ii) treatment with aqueous TFA in methylene chloride, which gave **12-18** in one step (96%). Jones oxidation generated γ-lactam **12-19**; interestingly, the primary alcohol was oxidized only to the aldehyde, which was attributed to severe steric hindrance circumventing the formation of the bulky chromate ester. Further oxidation was required to create the precursor for downstream β-lactone formation and was achieved using NaClO_2_, with subsequent esterification to methyl ester **12-20** in 44% overall yield from **12-16**. Protection of the tertiary alcohol and oxidative cleavage of the vinyl group with OsO_4_ and NaIO_4_ gave aldehyde **12-22** in preparation for reaction with cyclohex-2-enylzinc chloride as described by Corey’s group [39]; this gave **12-23** as the sole product in 90% yield. Unveiling of the corresponding triol was executed with BCl_3_ to afford Corey’s intermediate **12-24**, thereby completing the formal synthesis of **1**.

## 7. Closing Remarks

The novel structure and biological activity of **1** have inspired scientists from a variety of disciplines to target the salinosporamides for optimal production and analoging using traditional fermentation, industrial microbiology, classic natural products chemistry and semi-synthesis, total synthesis, and bioengineering. The resulting compounds have provided important insights into SAR, with the majority of structural modifications centered around P1 and P2. The P2 analogs have been excellent subjects for crystallographic studies in complex with the 20S proteasome, which together with SAR, firmly established the role of the leaving group in the mechanism of irreversible binding and prolonged duration proteasome inhibition *in vitro* and *in vivo.* Mutasynthesis offers a powerful technique to generate new P1 analogs, and has been complemented by total and semi-synthetic approaches. Clearly, the potential to generate proteasome subunit specific inhibitors exists, but apparently remains unfulfilled at the time of writing. With respect to the total synthesis of the parent natural product **1**, its dense functionality has attracted the attention of some of the most prestigious laboratories in the world. The growing number of total and formal syntheses promises the inevitable convergence to streamlined processes. However, in a powerful demonstration of industrial marine microbiology, *S. tropica* remains the formidable champion of robust production of **1**, fueling extensive preclinical studies and ongoing clinical trials, and demonstrating that the “supply issue” often associated with natural products can be overcome. Indeed, it is the parent natural product that is currently under clinical evaluation in patients with hematological and solid tumor malignancies. The remarkable progress demonstrated over a period of 7 years since the first publication of **1** by Fenical and coworkers anticipates new frontiers of achievement in the future of this important class of proteasome inhibitor. Beyond the current landscape of cancer therapeutics, the application of β-lactone proteasome inhibitors in the fight against microbial pathogens [7,8] is on the horizon.

## Figures and Tables

**Table 1:** Structures of **1** and P2 analogs, methods of production, and IC_50_ values for inhibition of CT-L activity of purified 20S proteasomes.

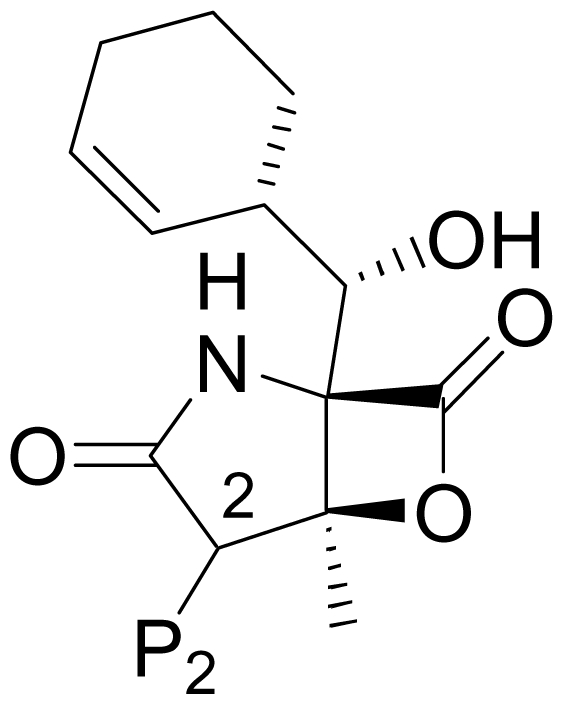				
Str #	Compound Name(s)	P2	CT-L (IC_50_, nM)^a^	Source/Method of Production
**1**	Salinosporamide A NPI-0052	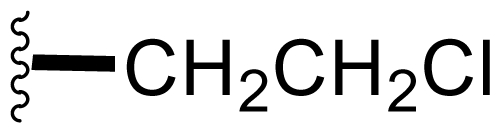	2.5 ± 1.2 [61] 2.0 ± 0.3 [61]^b^ 3.5 ± 0.3 [13]^c^	Natural Product [15] Total Synthesis [39–45] Formal Synthesis [46–50]
**5**	Salinosporamide B NPI-0047	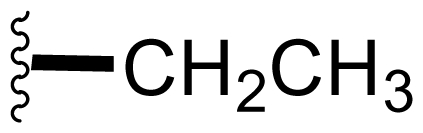	26 ± 6.7 [61]	Natural Product [51] Directed Biosynthesis [55,56,Table 4] Modified Media [54,58,Table 4]
**8**	Salinosporamide D	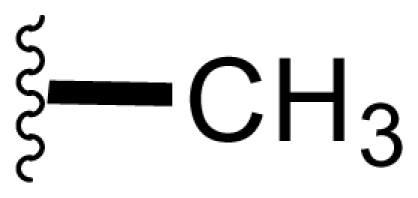	7.5 ± 0.6 [61]	Natural Product [52] Directed Biosynthesis [Table 4] Modified Media[Table 4]
**9**	Salinosporamide E	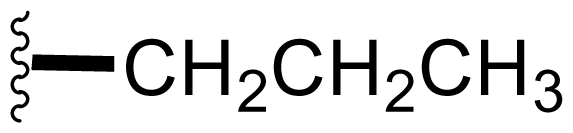	24 ± 5 [34]	Natural Product [52] Semi-synthesis [34] Directed Biosynthesis ± Modified Media [Table 4]
**10**	Salinosporamide F	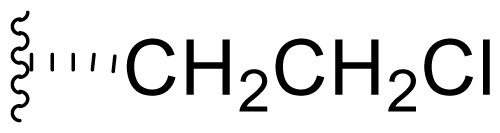	330 ± 20 [34]	Natural Product [34,52]
**11**	Salinosporamide G	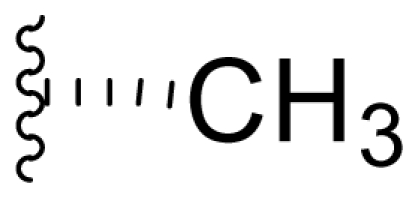	3200, 3300 [59]	Natural Product [52]
**16**	Salinosporamide H	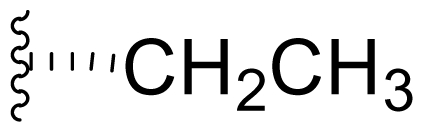	1400 [59]	Byproduct of directed biosynthesis of **15** [52]
**15**	Bromosalinosporamide	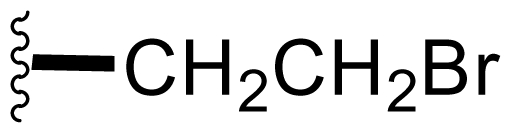	2.6 ± 0.4 [34]	Directed Biosynthesis [34,52,54] + Modified Media [Table 4]
**42**	Iodosalinosporamide	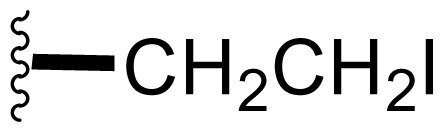	2.8 ± 0.5 [34]	Semi-synthesis [34]
**17**	Fluorosalinosporamide	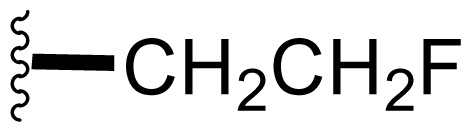	9.2 ± 10.2 [61] 1.5 ± 0.05 [63]^b^	Semi-synthesis [61] Mutasynthesis [63,66] Directed Biosynthesis in Modified Media [Table 4]
**43**	Azidosalinosporamide	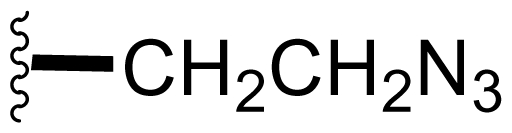	7.7 ± 1.5 [34]	Semi-synthesis [34]
**45**	Thiocyano-salinosporamide	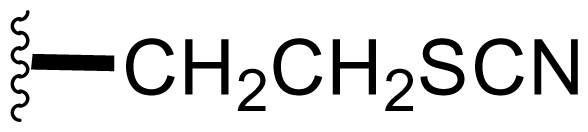	3.4 ± 0.2 [59]	Semi-synthesis [59]
**41**	Hydroxy-salinosporamide	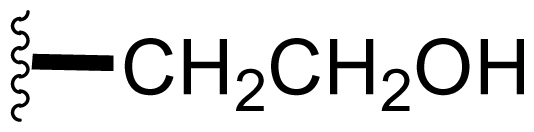	14 ± 1.5 [61]	Semi-synthesis [34,61]
**44**		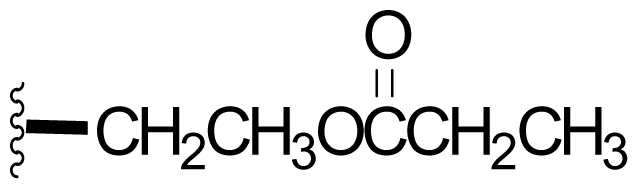	7,3 [59]	Semi-synthesis [59]
**46**	Mesylsalinosporamide	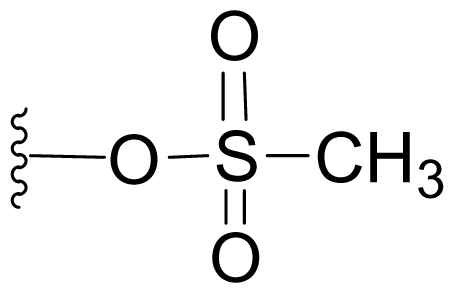	4.3 ± 0.8 [61]	Semi-synthesis [61]
**47**	Tosylsalinosporamide	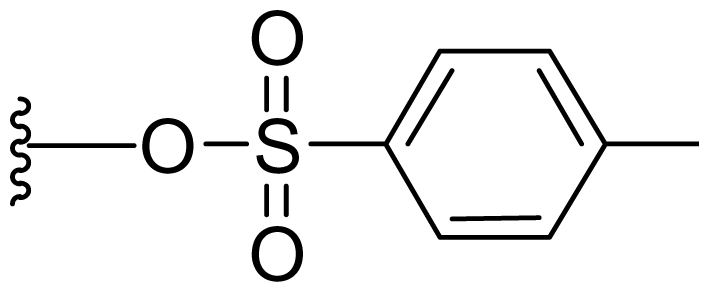	2.5 ± 0.4 [61]	Semi-synthesis [61] Total synthesis [61]
**48**	Dansylsalinosporamide	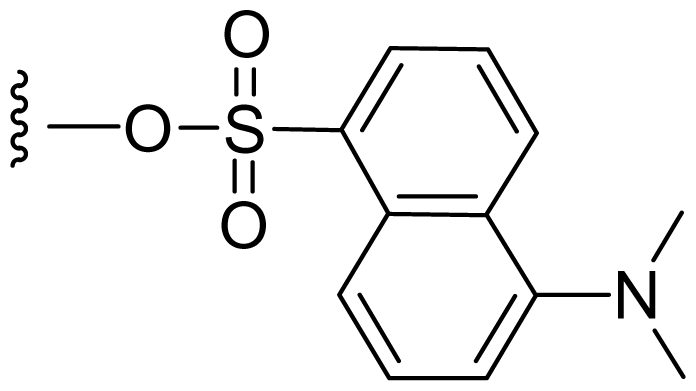	3.0 ± 0.5 [61]	Semi-synthesis [61]

a Purified rabbit 20S proteasomes, unless otherwise indicated. Where n ≥ 3, the mean IC_50_ value ± standard deviation is presented; where n < 3, results of individual experiment(s) are shown.

b Purified yeast 20S proteasomes.

c Purified human 20S proteasomes.

**Table 2:** Structures of **1** and P1 analogs, methods of production, and IC_50_ values for inhibition of CT-L activity of purified 20S proteasomes.

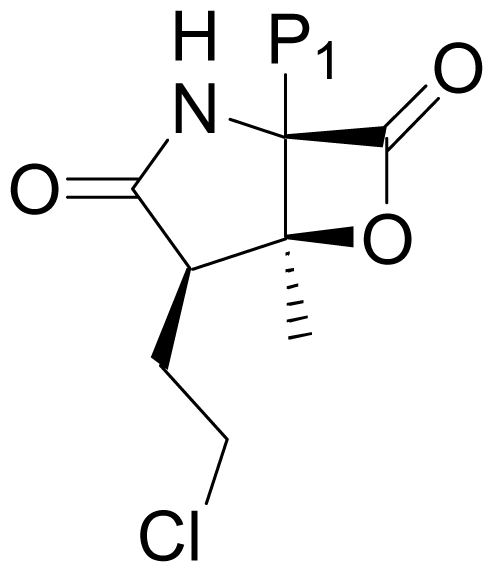				
Str #	Compound Name(s)	P1	CT-L (IC_50_, nM)^a^	Source/Method of Production
**1**	Salinosporamide A NPI-0052	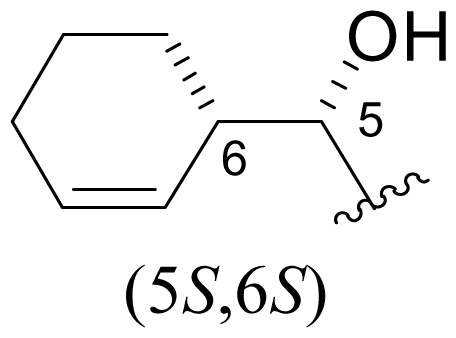	2.5 ± 1.2 [61] 2.0 ± 0.3 [61]^b^ 3.5 ± 0.3 [13]^c^	Natural Product [15] Total Synthesis [39–45] Formal Synthesis [46–50]
**13**	Salinosporamide J	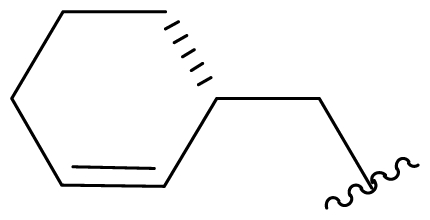	52 ± 2 [52]	Natural Product [52]
**36**	C-5-*epi*-salinosporamide	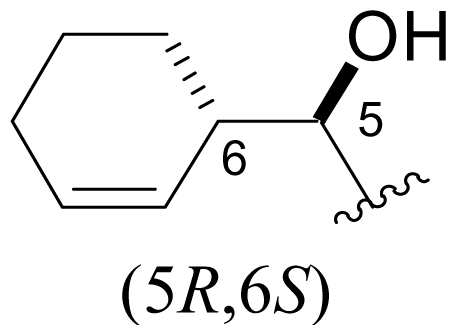	> 20000 [34]	Semi-synthesis [34,60] Total synthesis [41]
**35**	Keto-salinosporamide	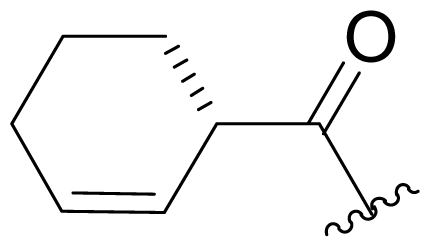	8200 ± 600 [34]	Semi-synthesis [34,60] Total synthesis [41]
**18**	NPI-2056 Salinosporamide X1	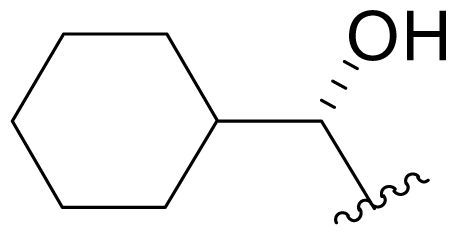	20 ± 3 [34] 27.5 ± 3.7 [65]^b^	Semi-synthesis [34] Mutasynthesis [64,65]
**19**	Salinosporamide X7	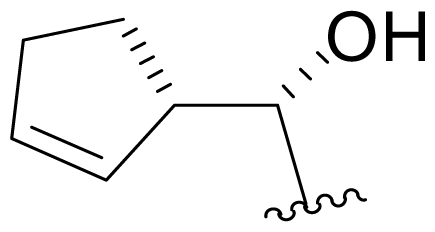	2.2 ± 0.1 [65]^b^	Mutasynthesis [65]
**20**	Salinosporamide X2	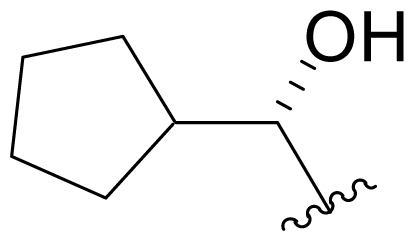	9.3 ± 1.6 [65]^b^	Mutasynthesis [64,65]
**21**	Salinosporamide X3	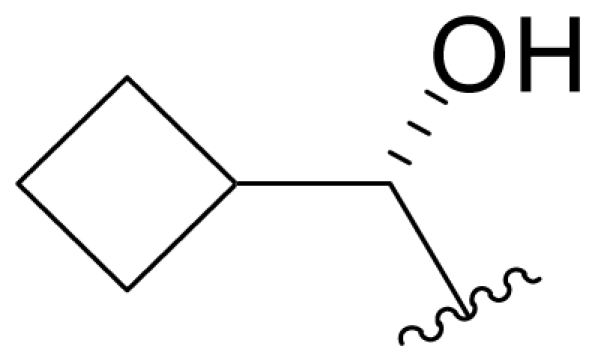	93.4 ± 4.3 [65]^b^	Mutasynthesis [65]
**14**	Antiprotealide	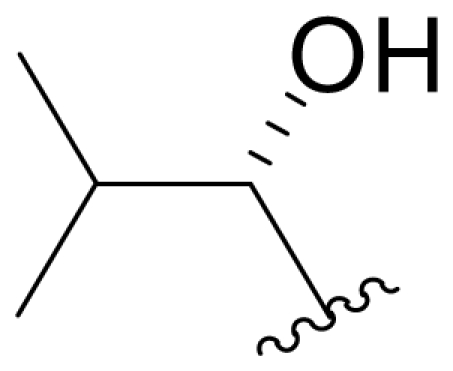	31 ± 5 [53]^d^ 27 ± 2 [53]^e^ 101 ± 15 [65]^b^	Natural Product [53] Total Synthesis [71,72] Mutasynthesis [64,65] Directed Biosynthesis [Table 4]
**22**	Salinosporamide X5	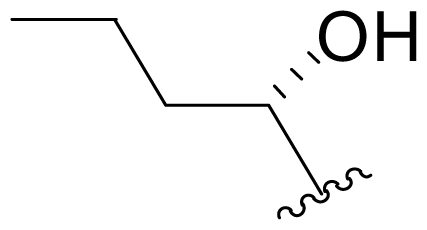	245 ± 38 [65]^b^	Mutasynthesis [65]
**23**	Salinosporamide X6	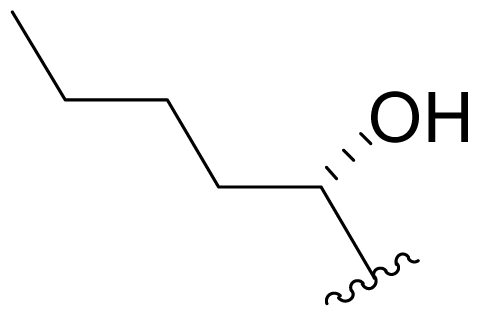	132 ± 19 [65]^b^	Mutasynthesis [65]
**24**	Salinosporamide X4	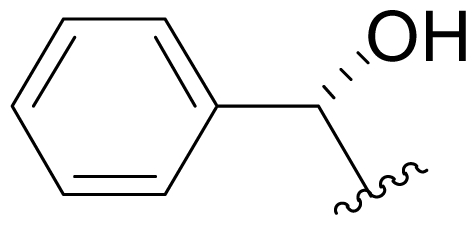	1029 ± 419 [65]^b^	Mutasynthesis [65]
**37**	NPI-2060	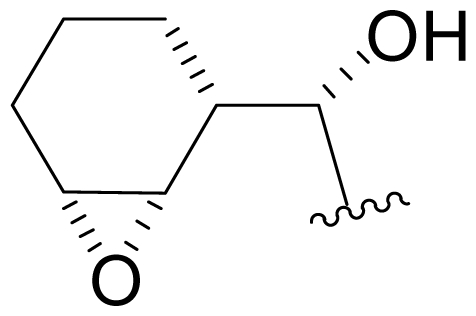	6.3 ± 0.6 [34]	Semi-synthesis [34]
**38**	NPI-2061	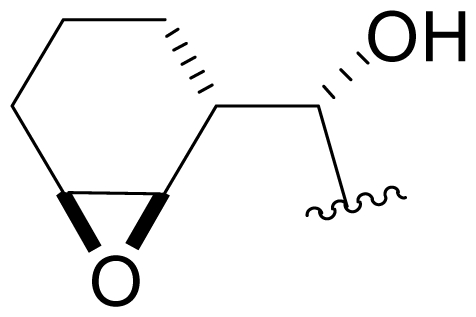	91 ± 8 [34]	Semi-synthesis [34]
**39**	NPI-2064	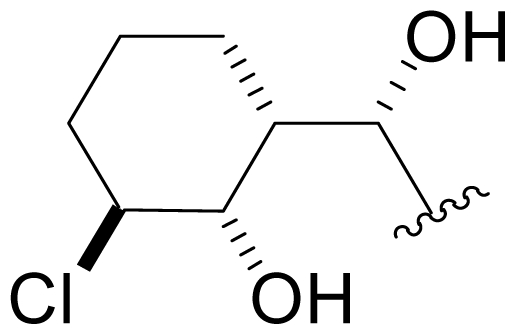	8200 ± 3000 [34]	Semi-synthesis [34]
**34**	NPI-2157	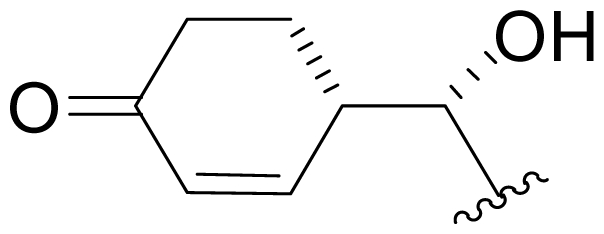	NR	Degradation [Scheme 1] Semi-synthesis [Scheme 2]
**40**	NPI-2167	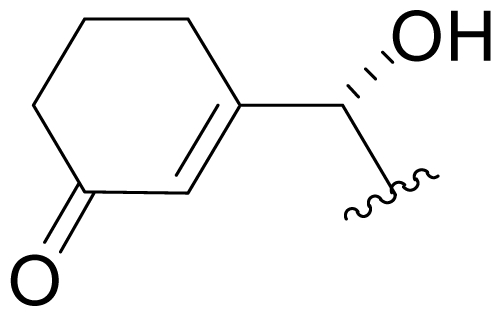	NR	Semi-synthesis [Scheme 2]

NR: Not reported;

a Purified rabbit 20S proteasomes, unless otherwise indicated. The mean IC_50_ value ± standard deviation is presented, except for compound **36**, for which the mean of 2 individual experiments is shown.

b Purified yeast 20S proteasomes.

c Purified human 20S proteasomes.

d Natural product purified from *S. tropica* extracts.

e Synthetic product.

**Table 3:** Fermentation yield improvement of **1** in shake flask and laboratory fermentor.

Improvement step	Improvement parameters	Shake flask (mg/L)	Fermentor (mg/L)
–	Original condition	4	–
1	Effect of resins	70	25
2	Length and timing of seed, production and resin addition cycle	120	120
3	Media formulation	220	220
4	Single colony isolation	330	330
5	Statistical design media optimization	450	360

**Table 4:** Production of salinosporamides (mg/L) by precursor-directed biosynthesis using wild-type *S. tropica* strain NPS21184.

Condition	Precursor	1 Chloro	15 Bromo	17 Fluoro	14 Antiprotealide	5 Ethyl	8 Methyl	9 Propyl
1 [53]	None (NaCl-based medium)	277	0	0	3.0	4.4	0.15	0.11
2 [54]	NaBr (NaBr-based medium)	1.2	19.4	0	0	80.3	0	0
3	None (Na_2_SO_4_-based medium)	53	0	0	ND	7.3	0.34	0.18
4	1.5% NaBr (Na_2_SO_4_-based medium)	18.7	73.3	0	0	22.3	0	0
5	0.025% 5′-FDA (Na_2_SO_4_-based medium)	48.1	0	55.8	ND	2.9	Trace	Trace
6	1% Valerate (Na_2_SO_4_-based medium)	45	0	0	ND	8.3	0.12	145
7	1% Propionate (NaCl-based medium)	222	0	0	0	5.2	4.81	0
8 [55]	1% Butyrate (NaCl-based medium)	189	0	0	ND	19.0	0	0
9	1% Valerate (NaCl-based medium)	131	0	0	ND	5.0	0.15	121
10	1% Valine (NaCl-based medium)	177	0	0	0	16.7	0	0
11	1% Leucine (NaCl-based medium)	112	0	0	11	11	0.43	0.23
12	1% Isoleucine (NaCl-based medium)	71	0	0	0	6.6	4.63	0

ND = Not determined.
